# Nitride-Based Quantum Structures in Optoelectronics—A Survey of Colors

**DOI:** 10.3390/ma19143088

**Published:** 2026-07-17

**Authors:** Iza Gorczyca, Tadek Suski, Piotr Perlin, Grzegorz Staszczak

**Affiliations:** Institute of High Pressures Physics, UNIPRESS, 01-142 Warsaw, Poland

**Keywords:** nitride quantum structures, InGaN, AlGaN, external quantum efficiency, light-emitting diodes, laser diodes, optoelectronics

## Abstract

Modern optoelectronic devices, such as light-emitting diodes (LEDs) and laser diodes, rely on nitride-based (GaN, AlN, and InN) quantum structures, which underpin current technologies. These systems enable emission across a broad spectral range from ultraviolet to infrared with properties tunable via composition, strain, and quantum confinement. This review summarizes progress in the performance of nitride emitters across the full spectral range, with particular emphasis on the evolution of external quantum efficiency (EQE). Nitride emitters are primarily based on InGaN quantum wells, while AlGaN quantum wells are used for ultraviolet operation. Device performance is governed by the intrinsic properties of these structures, which also determine key physical challenges across different wavelength regions. Blue InGaN LEDs achieve the highest efficiencies (~60–80% EQE), while green devices are limited to ~20–35% due to the “green gap,” with further reduction (~5–20%) toward longer wavelengths. In the ultraviolet, AlGaN-based emitters exhibit lower performance due to material and structural challenges, although steady progress is being made. Special attention is given to mechanisms limiting EQE, including efficiency droop in the green–red region, and ongoing efforts to mitigate these effects. Finally, perspectives for future applications of [ are outlined.

## 1. Introduction

Nitride-based quantum structures (QSs) form the foundation of optoelectronic devices such as light-emitting diodes (LEDs) and laser diodes (LDs), operating across a broad spectral range from the ultraviolet (UV) to the infrared (IR). Their emission properties can be engineered through the choice of quantum-structure type, composition, and material design.

Most semiconductor light emitters are based on III–V compounds; however, conventional arsenide- and phosphide-based systems are limited in achieving sufficiently wide band gaps for efficient emission in the green–blue spectral range. Although alloying materials such as GaAs with Al or P increases the band gap, these alloys tend to become indirect beyond certain compositions, significantly reducing radiative efficiency. Consequently, these systems are largely restricted to the red and (part of the) yellow spectral range.

To reach higher photon energies, wide-band gap II-VI semiconductors, particularly Zn-based compounds, have been extensively investigated. While these materials enable blue–green emission and even lasing, their practical use is limited by poor reliability, including short device lifetimes, high operating voltages, and the lack of lattice-matched substrates.

**Figure 1:**
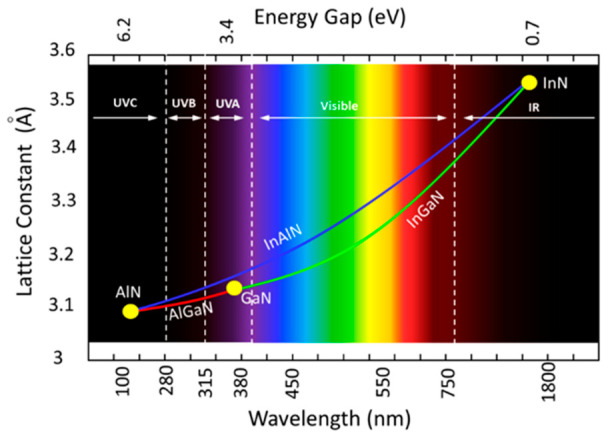
Schematic illustration of the light-emission spectral range potentially achievable using nitride-based QSs.

In contrast, GaN and its alloys with In (InGaN) and Al (AlGaN) offer a much wider band-gap range, enabling emission from the deep UV to the near-IR. InGaN-based LEDs and LDs cover the visible spectrum, particularly the blue–green region, while AlGaN-based structures extend operation into the UV. This wide tunability makes the III-nitride system uniquely versatile for both incoherent and coherent light sources. Nowadays, III-nitride semiconductors constitute one of the most important material systems in modern optoelectronics. Quantum wells based on III-nitride materials form the active regions of LEDs and LDs, which underpin solid-state lighting, display technologies, and a wide range of emerging photonic applications. Figure 1 schematically illustrates the spectral range accessible with nitride-based quantum structures.

For over three decades, research on III-nitrides has focused on improving luminous efficiency, initially targeting high-efficiency blue emission and later extending across the visible spectrum [1,2]. Progress has been driven by advances in epitaxial growth, doping, and defect reduction, as well as by the transition from large-area LEDs to low-dimensional structures, as micro- and nano-LEDs.

The development of visible-light LEDs has transformed modern lighting, especially through white sources based on blue InGaN emission with phosphors like YAG:Ce. Now exceeding 10 billion units annually, LED bulbs have largely replaced incandescent lighting, significantly reducing energy consumption. Their impact comes from high luminous efficacy (>150 lm/W, up to 250 lm/W [3]), along with easy control, smart-system compatibility, and tunable color temperatures. White LEDs are widely used for backlighting in displays (monitors, TVs, and mobile devices), reaching several billion units annually, and are also key in automotive lighting (headlights, daytime running lights, and interiors). High-power red, amber, and green LEDs are essential for traffic signals and large-scale signaling systems. Blue and green LEDs underpin display technologies (RGB and micro-LED) and architectural lighting, while blue LEDs additionally enable white light via phosphors and are used in communication, medical illumination, and fluorescence sensing.

UV LEDs further broaden applications: UVA (320–400 nm) are used in curing, fluorescence detection, and 3D printing; UVB (280–320 nm) in specialized medical treatments such as phototherapy; and UVC (200–280 nm) in disinfection of water, air, and surfaces by inactivating microorganisms through DNA and RNA damage.

Although LEDs dominate the market, LDs remain essential due to their high coherence and optical power density. In the UV–blue range, nitride-based LDs are used in optical data storage (e.g., Blu-ray Disc), digital printing, high-resolution projection, and advanced RGB displays. Green LDs are particularly important for laser projection, AR/VR systems, and high-brightness displays, enabling wide color gamut and high efficiency. Their use also extends to spectroscopy, biomedical applications, and optical metrology, where directional, monochromatic light is required.

Despite progress toward longer wavelengths, efficient red-emitting nitride LDs have not been achieved. This is mainly due to material challenges, including the high indium content required, which degrades crystal quality, enhances internal electric fields, and reduces electron-hole overlap. As a result, red laser diodes are typically based on alternative systems such as AlGaInP.

Nitride light emitters are based on QSs, including layered systems and low-dimensional forms such as nanowires and quantum dots (QDs).

**Layered QSs** comprise single and multi-quantum wells (SQWs, MQWs) and superlattices (SLs), with properties determined by layer thickness and crystallographic orientation. MQWs consist of isolated QWs separated by thick barriers, whereas SLs are formed by thin, strongly coupled layers, leading to wavefunction overlap and miniband formation. Thus, SLs can be regarded as short-period MQWs with strong inter-well coupling.

**Low-dimensional structures**—such as micro-/nanostructures, QDs, and nanowires—are promising for LEDs and LDs due to suppressed internal electric fields, reduced defect densities, and enhanced indium incorporation. Nanowires support efficient carrier transport, while QDs, with discrete energy levels, exhibit size-dependent emission. Nitride QDs offer strain relaxation, reduced confinement [4], strong carrier localization that suppresses non-radiative recombination [5,6], large exciton binding energies [7,8], and potential mitigation of “efficiency droop” and the “green gap” [9,10].

However, their properties are highly size-dependent, and their quantum efficiency is typically lower than that of MQWs. The growth of InGaN QDs remains challenging due to thermal instability, often limiting room-temperature (RT) emission. Nevertheless, single QDs or nanowires can act as single-photon emitters and are also used in larger-scale devices (e.g., as QD layers), combining micro- and macro-scale features.

Nitride light emitters are mainly based on InGaN QWs, with AlGaN QWs used for UV emission, and their performance is governed by intrinsic material properties.

We start the review with blue InGaN emitters, historically the first and still the most efficient. Toward shorter wavelengths, UV AlGaN emitters remain less efficient due to material limitations, although continuous progress is being made. Next, we move from blue toward the green–red spectral range, where efficiency decreases because of difficulties in growing high quality, high-indium InGaN QWs. Nevertheless, recent advances in high-In-content InGaN have brought efficient red emission closer to realization, supporting the development of monolithic RGB devices. The review concludes by outlining future directions, including new concepts and emerging systems such as h-BN and hybrid nitride/oxide heterostructures.

## 2. Quantum Wells in Nitride Light Emitters

From a physics perspective, nitride devices remain challenging due to their complex material properties. Although the general concept of QWs in nitride emitters is similar to that in GaInAsP systems, important differences arise. Nitride semiconductors have a hexagonal wurtzite (wz) structure without inversion symmetry, which—together with strong bond iconicity—leads to significant spontaneous and piezoelectric polarization [11,12].

### 2.1. Quantum Confined Stark Effect

**Figure 2:**
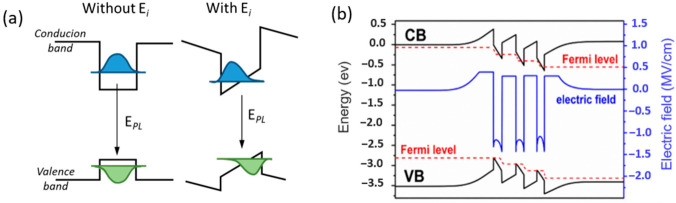
(**a**) Schematic illustration of QCSE for single QW without (**left**) and with (**right**) internal electric field, E_i_. Where E_PL_ denotes the photoluminescence energy, (**b**) calculated band profiles of InGaN/GaN MQW along c-axis of wz structure.

In InGaN/GaN QWs, differences in composition and strain between compressively strained InGaN and nearly strain-free GaN generate polarization discontinuities at the heterointerfaces. These discontinuities induce fixed interface charges and strong internal electric fields, which tilt the conduction and valence band edges and spatially separate electron and hole wave functions, giving rise to the quantum-confined Stark effect (QCSE). The QCSE is a characteristic feature of wurtzite (WZ) QSs and strongly influences their optical properties. Figure 2a schematically illustrates the QCSE, while Figure 2b presents its impact on the electronic band structure, utilizing calculated band profiles of InGaN/GaN MQWs.

QCSE is not unique to III-N semiconductors; it also occurs in GaAs/InGaAs structures grown along the (111) direction [13], though much more weakly, whereas in InGaN QWs internal fields can reach 1–3 MV/cm [14]. It modifies optical properties by increasing carrier lifetimes (from sub-ns to tens or hundreds of ns [15]) and causing a redshift of emission. As a result, emission depends not only on composition but also on well width and polarization differences, with recombination resembling spatially indirect donor–acceptor pair transitions.

The QCSE has long been considered detrimental to device efficiency, since the optical transition matrix element is proportional to the electron-hole wavefunction overlap:

(1) $$M_{cv}\approx \langle u_{c}|\hat{e}\cdot p|u_{v}\rangle \int \Psi_{e}^{*}(r)\Psi_{h}(r)d^{3}r$$

where $u_{c},u_{v}$ are Bloch functions and $\Psi_{e},\Psi_{h}$ are electron and hole wavefunctions. The radiative efficiency is given by:

(2) $$\eta_{rad}=\frac{1/\tau_{r}}{1/\tau_{r}+1/\tau_{nr}}$$

Thus, $\tau_{r}\ll \tau_{nr}$ would suggest low efficiency. However, despite long recombination times in InGaN QWs, high radiative efficiency is observed. This apparent paradox was resolved around 2013 by Kioupakis et al. [16], who showed that both radiative and non-radiative recombination rates are similarly affected by the internal electric field associated with QCSE.

Experimental studies (e.g., Marona et al. [15])) confirmed that both $\tau_{r}$ and $\tau_{nr}$ increase with increasing field. Nevertheless, the reduced overlap $M_{cv}$ remains detrimental for LDs, since the optical gain is proportional to $|M_{cv}{|}^{2}$. As the electron-hole wavefunction overlap decreases, the optical gain is reduced, which is critical because the gain must overcome resonator losses for lasing to occur [17]. To mitigate the QCSE, two main strategies are used: growth of QWs on nonpolar or semipolar crystallographic planes and engineering of staggered QW structures to enhance carrier overlap.

### 2.2. Nonpolar and Semipolar Crystallographic Planes

In wz nitrides, polarization discontinuity strongly depends on crystal orientation, ranging from fully polar c-plane (0°) to nonpolar m-plane (90°). Semipolar orientations have been widely studied, notably by the University of California, Santa Barbara group [18], demonstrating promising device performance. However, their practical use is limited by high oxygen incorporation during growth [18,19], strain relaxation via dislocation formation [19], and the limited availability of large-area (≥2-inch) substrates.

#### Staggered QWs

**Figure 3:**
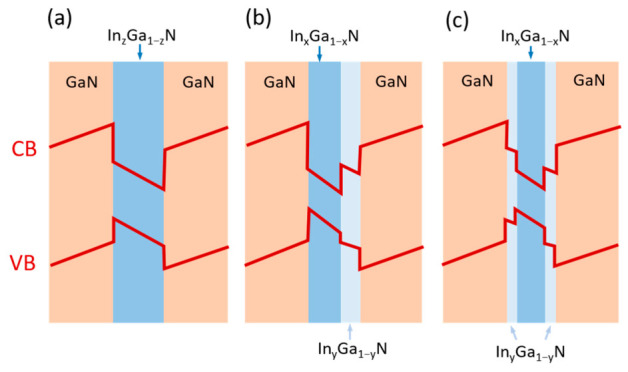
Schematic of the (**a**) conventional In_x_Ga_1−x_N/GaN QW, (**b**) two-layer staggered In_x_Ga_1−x_N/In_y_Ga_1−y_N QW and (**c**) three-layer staggered In_x_Ga_1−x_N/In_y_Ga_1−y_N/In_x_Ga_1−x_N QW structures. Based on Figure 2.20 of Ref. [20].

An alternative approach is quantum well (QW) engineering to enhance electron–hole overlap using staggered QWs. These structures consist of ultrathin layers forming shallow and deep potential wells, in various configurations (Figure 3). In staggered InGaN QWs, the overlap integral can increase by up to a factor of two, significantly reducing the QCSE. Such designs are widely used, although their implementation depends on the overall LED or LD architecture.

A key challenge of InGaN QWs is achieving pseudomorphic growth at high indium content, due to the large lattice mismatch (~11.6%) between GaN and InN. As indium incorporation increases, strain energy rises, leading to “composition pulling” [21], which limits further incorporation. Above ~27% In, growth tends to become three-dimensional, hindering pseudomorphic layers [22]. Even a monolayer of InN on GaN yields only ~30% average incorporation [23,24], highlighting strong strain constraints. These effects are also linked to spinodal decomposition and Stranski–Krastanov growth instability [25]. Approaches to mitigate this include relaxed InGaN buffers [26] and compliant templates such as porous GaN [27].

### 2.3. Sources of Non-Radiative Recombination

Since most nitride LEDs are grown on sapphire, they exhibit high threading dislocation densities (~10^8^–10^9^ cm^−2^). While such defects would typically reduce radiative efficiency, InGaN devices remain highly efficient. This paradox was first addressed by Chichibu [28], who attributed it to carrier localization in potential minima caused by compositional fluctuations limiting diffusion to dislocations. However, reported long carrier diffusion lengths (μm scale) [29,30] are not fully consistent with this model.

An alternative explanation by Hangleiter et al. [31] suggests that threading dislocations form V-shaped pits with sidewall QWs of a higher effective band gap, creating barriers that prevent carrier capture and account for both high efficiency and long-range transport.

In addition to extended defects such as dislocations, intrinsic non-radiative processes also strongly affect InGaN QW efficiency. A key issue is the efficiency droop in InGaN LEDs [32], i.e., reduced radiative efficiency at high current densities. Initially attributed to electron leakage into p-type layers [32], it is now mainly linked to Auger recombination in the QWs [33]. Because conventional Auger processes are expected to be weak in wide-bandgap materials, mechanisms such as phonon-assisted and disorder-enhanced Auger recombination have been proposed [34]. In particular, Shockley–Read–Hall (SRH) recombination via defect centers is significant. Defects formed during metalorganic vapor phase epitaxy (MOVPE) growth, such as nitrogen vacancies and $V_{N}-V_{In}$ divacancies, have been identified as efficient SRH centers [35]. Other defects, including $V_{Ga}$ and complexes with H and O, are also considered important recombination centers in InGaN [36].

### 2.4. Al_x_Ga_1−x_N/Al_y_Ga_1−y_N and AlInGaN/AlGaN QWs for UV Emitters

**Figure 4:**
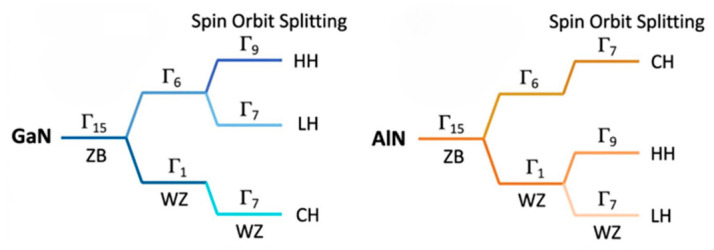
Schematic of the valence band sub-bands in GaN and AlGaN.

Since the bandgap of GaN at RT corresponds to ~365 nm emission, shorter-wavelength emitters require AlGaN or quaternary AlInGaN. AlGaN can be grown over the full composition range without phase separation [37], but unlike InGaN, its radiative efficiency is more sensitive to dislocation density—a trend also observed in low-In-content InGaN [38]. In InGaN/GaN QWs, strong piezoelectric polarization generates internal fields of ~1–3 MV/cm, causing pronounced QCSE and reduced electron-hole overlap. In contrast, AlGaN/AlGaN QWs have smaller lattice mismatch, leading to weaker piezoelectric effects and a relatively larger role of spontaneous polarization. Thus, polarization balance in AlGaN structures is more sensitive to composition and strain. While both systems exhibit strong polarization effects, InGaN/GaN is typically piezoelectric-dominated, whereas in AlGaN/GaN the spontaneous and piezoelectric contributions are more comparable [39,40]. The valence band in wz nitrides consists of three sub-bands: heavy hole (Γ_9_), light hole (Γ_7_), and crystal-field split-off hole (Γ_7_) (see Figure 4).

In Al_x_Ga_1−x_N alloys, increasing Al content causes valence band reordering due to competition between crystal-field splitting (Δcf) and spin-orbit interaction (Δso). In GaN (Δcf > 0), the top valence band is Γ_9_ (HH), while in AlN (Δcf < 0) it is Γ_7_ (CH). The transition occurs at x ≈ 0.6–0.7 (strain-dependent).

This inversion changes the emission character from TE to TM polarization. In c-plane devices, TM light is emitted along the growth direction and is poorly extracted, reducing efficiency. It also alters transition matrix elements, lowering radiative recombination—an effect particularly important for deep-UV AlGaN emitters [41,42].

## 3. Efficiency Parameters of III-Nitride LEDs and LDs

The performance of III-nitride emitters is commonly evaluated using the external quantum efficiency (EQE), defined as the ratio of emitted photons to injected electrons:

(3) $$EQE=\eta_{inj}\times IQE\times \eta_{ext}$$

where:

$\eta_{inj}$ is the carrier injection efficiency, i.e., the fraction of injected carriers that reach the active region; *IQE* is the internal quantum efficiency, i.e., the fraction of carriers recombining radiatively within the active region; and $\eta_{ext}$ is the light extraction efficiency, i.e., the fraction of internally generated photons that escape from the device. The internal quantum efficiency (IQE) is given by:

(4) $$IQE=\frac{R_{rad}}{R_{rad}+R_{nonrad}}$$

where:

$R_{rad}$ is the radiative recombination rate; $R_{nonrad}$ is the non-radiative recombination rate. 

A more comprehensive metric is the wall-plug efficiency (WPE), defined as the ratio of the optical output power P_out_ to the supplied electrical power:

(5) $$WPE=\frac{P_{out}}{P_{el}}=\frac{P_{opt}}{\left(I\times V\right)}$$

where:

P_el_ is the electrical input power; I is the injection current; V is the operating voltage.

For LEDs, the relationship between WPE and EQE can be expressed as:

(6) $$WPE=EQE\frac{h\nu }{qV}$$

where hν is the photon energy; q is the elementary charge.

This expression shows that WPE depends both on carrier-to-photon conversion efficiency and on electrical efficiency. Consequently, devices with high EQE may still exhibit low WPE because of excessive voltage drop, series resistance, leakage currents, or other parasitic losses.

For III-nitride LEDs, IQE is commonly described by the ABC recombination model:

(7) $$IQE=\frac{Bn^{2}}{An+Bn^{2}+Cn^{3}}$$

where: *A* is the Shockley–Read–Hall (SRH) non-radiative recombination coefficient associated with defect-related recombination; *B* is the radiative recombination coefficient; *C* is the Auger recombination coefficient associated with high-carrier-density losses; and *n* is the carrier concentration in the active region.

Substituting the ABC expression into the EQE equation yields

(8) $$EQE=\eta_{inj}\eta_{ext}\frac{Bn^{2}}{An+Bn^{2}+Cn^{3}}$$

This expression is widely used to evaluate the performance of III-nitride laser diodes and to analyze the influence of carrier leakage, non-radiative recombination, and optical cavity losses on device efficiency.

A formulation analogous to the LED ABC model may be written as:

(9) $$\eta_{d}=\eta_{inj}\times \eta_{int}\times \eta_{ext}$$

where: $\eta_{inj}$ is the carrier injection efficiency; $\eta_{int}$ is the internal quantum efficiency, representing the fraction of injected carriers contributing to stimulated emission; and $\eta_{ext}$ is the photon extraction efficiency, which is determined by cavity losses and mirror transmission. The extraction efficiency is commonly expressed as:

(10) $$\eta_{ext}=\frac{\alpha_{m}}{\alpha_{i}+\alpha_{m}}$$

where $\alpha_{i}$ is the internal optical loss coefficient; $\alpha_{m}$ is the mirror loss coefficient.

Consequently, the differential external quantum efficiency can be written as:

(11) $$\eta_{d}=\eta_{inj}\eta_{int}\frac{\alpha_{m}}{\alpha_{i}+\alpha_{m}}$$

This relation is widely used to evaluate III-nitride laser diodes and quantify the influence of optical losses and carrier injection efficiency.

For completeness, an ABC-type expression analogous to that used for LEDs may be written as:

(12) $$\eta_{int}=\frac{Bn^{2}}{An+Bn^{2}+Cn^{3}+R_{leak}}$$

where $R_{leak}$ represents carrier leakage losses. Above the threshold, however, stimulated emission dominates, so LD analyses are usually based on the differential efficiency expression rather than the LED EQE formula.

A few comments on the physical basis of various efficiency parameters are useful at this point.

In LEDs, the light extraction efficiency, $\eta_{ext}$, is related to the fact that the light generated in the QWs must escape from a semiconductor the refractive index of which is much higher than that of the surrounding medium. This leads to the formation of an escape cone defined by the critical angle for total internal reflection. For GaN (n≈2.4−2.5 in the visible range), the escape angle is only about $23^{\circ }$ with respect to the surface normal. As a result, only a small fraction of the generated photons can leave the semiconductor directly. Typically, $\eta_{ext}$ can be increased by surface roughening, patterned substrates, photonic structures, or chip shaping, all of which help redirect photons into the escape cone.

The injection efficiency, $\eta_{inj}$, describes the fraction of the electrical current that is injected into the QWs, where radiative recombination takes place. The remaining current contributes to parasitic recombination processes outside the active region and therefore does not generate useful light. In nitride-based structures, an electron-blocking layer (EBL) is commonly employed to suppress electron leakage from the active region and thus improve $\eta_{inj}$.

## 4. Nitride Light Emitters: From Blue to Ultraviolet (500-200)

LED and LD epitaxial structures differ fundamentally in both complexity and operation. InGaN-based LEDs typically employ relatively simple InGaN/GaN MQW structures designed for spontaneous emission over a broad area, without the need for optical confinement or a resonant cavity. In contrast, blue LDs require carefully engineered QWs, waveguides, cladding layers, and current confinement structures to achieve stimulated emission and optical gain. Consequently, their epitaxial structures are significantly more complex.

A standard GaN-based blue/violet LD typically consists of a sapphire substrate (or GaN in modern devices), n-AlGaN cladding layer, lower InGaN waveguide, MQW active region (usually 2–4 InGaN QWs), upper InGaN waveguide, AlGaN EBL, p-AlGaN cladding layer, and a heavily doped p-type contact layer. Together, these layers provide efficient carrier and optical confinement as well as stimulated recombination within the QWs.

**Figure 5:**
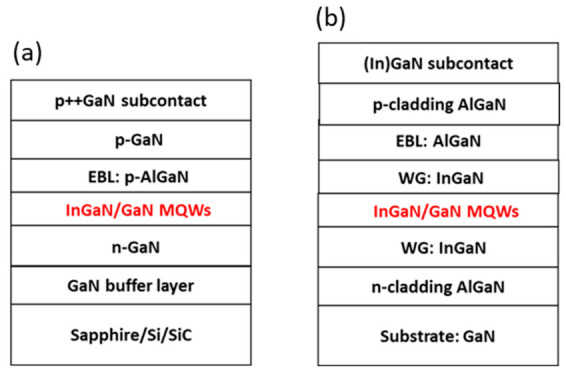
Schematic diagram of the epitaxial layers of InGaN-based blue (**a**) LEDs and (**b**) LDs. Active regions are marked in red.

Figure 5 schematically compares the epitaxial structures of a typical InGaN-based blue (a) LED and (b) laser diode (LD). The general device architecture is broadly applicable to most nitride light emitters. Across the visible spectral range, active regions are typically based on InGaN QWs, whereas AlGaN QWs are commonly employed for UV emission.

The main differences between individual devices arise from variations in layer composition and structural design optimized for specific operating wavelengths and device requirements. In the following, LEDs and LDs operating across successive spectral ranges are briefly described, with particular attention to the quantum structures forming the active region and to the external quantum efficiency (EQE) of the devices.

### 4.1. Blue–Violet (500-400)

#### 4.1.1. LEDs

In 1994, following the growth of high-quality InGaN films, Nakamura and coworkers [43] demonstrated candela-class, high-brightness InGaN/AlGaN blue LEDs suitable for commercial applications, with luminous intensities exceeding 1 cd. The active region consisted of a Zn-doped InGaN/GaN MQW. The devices exhibited a typical output power of ~1.5 mW and EQE of up to 2.7% at a forward current of 20 mA under RT operation.

In the same year Nakamura et al. [44] fabricated SQW LEDs emitting in the blue to green spectral range. The active region consisted of a single 2 nm thick InGaN/GaN QW. The blue LEDs exhibited an output power of 4 mW at a drive current of 30 mA and an EQE of 2.4% at 20 mA under RT operation. The electroluminescence (EL) peak was centered at 500 nm.

Following the initial demonstrations of InGaN/GaN blue LEDs, development in the 400–500 nm range rapidly progressed from proof-of-concept devices to highly efficient commercial LEDs. In the early 2000s, optimization of MQW active regions, barrier design, strain engineering, and light extraction enabled blue LEDs with EQEs exceeding 30–40%, later surpassing 50% in commercial devices.

Narukawa et al. [45,46,47] fabricated high-luminous-efficiency white LEDs using high-efficiency blue LEDs and YAG phosphors. The reported blue devices exhibited output powers of 47.1 and 42.2 mW with EQEs of 84.3% and 75.5%, respectively. The efficiency improvement was attributed to: (i) highly transparent indium tin oxide (ITO) electrodes (>95% transmittance at 450 nm), which serve as transparent p-type contact layers enabling uniform current injection while minimizing optical absorption, and (ii) a patterned sapphire substrate (PSS) that enhanced light extraction by reducing total internal reflection within the nitride layers. This indicated that simultaneous optimization of IQE and light extraction is essential for achieving very high EQE.

Parallel advances in micro-LED technology further exploited the high internal efficiency of InGaN, enabling efficient emitters for displays and visible-light communication. Collectively, these developments established III–V nitrides as the dominant material system for blue and violet light emitters.

Hurni et al. [48] reported violet-emitting III-nitride LEDs grown on bulk GaN substrates, employing a triangular volumetric flip-chip architecture. The device performance is optimized for operation at high current densities and elevated temperatures through specific design considerations for the epitaxial layers, light extraction efficiency, and electrical injection. They demonstrated that the use of bulk GaN substrates systematically enables improved performance over standard LED technology, including superior IQE, EQE, and electrical efficiency. The EQE reaches 80% at a wavelength of 415 nm.

Li et al. [49] demonstrated cascaded blue/green micro-LEDs with independently controlled junctions grown by metal-organic chemical vapor deposition (MOCVD). The devices integrate blue μLEDs, a tunnel junction, and green μLEDs, enabling independent blue, green, and combined emission. The blue (60 × 60 μm^2^) μLEDs exhibit a forward voltage of 4.1 V at 20 A/cm^2^, with peak EQEs of 42%. The emission peak is located at 518 nm, with a FWHM value of 19. These results demonstrate the potential of cascaded LEDs for monolithic full-color integration. Sheen et al. [50] demonstrated blue InGaN nano-LEDs with an EQE of 20.2 ± 0.6% at ~440 nm using sol–gel SiO_2_ surface passivation, which reduced surface recombination by passivating GaN dangling bonds.

Higher EQE values have been reported recently. Choi et al. [51] in 2024 investigated efficiency droop in blue and green GaN-based LEDs and LDs using identical epitaxial structures with different indium contents. The active region consisted of 2.0 nm InGaN QWs separated by 7.5 nm InGaN QBs. For the blue devices, the QWs contained 15% In, while the QBs contained 2% In. The resulting emission wavelengths were 430 nm for LEDs and 440 nm for LDs, with corresponding EQE values at 25 mA of approximately 91.9% and 44.7%, respectively.

#### 4.1.2. LDs

**Figure 6:**
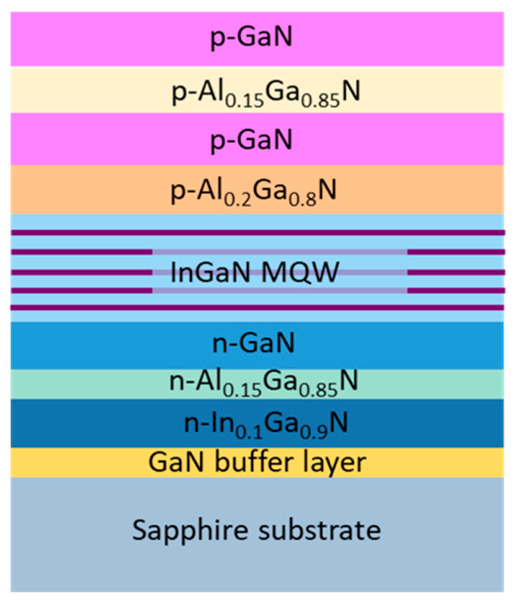
The epitaxial structure of the first InGaN MQW LD. Based on Figure 1 of Ref. [52]. Later in 1996, Nakamura’s group achieved the first CW operation of a violet InGaN LD [53].

The first nitride-based LDs, emitting at 417 nm, were demonstrated in 1996 by Shuji Nakamura and co-workers [52]. The devices were grown by MOVPE on sapphire substrates with an n-type GaN buffer layer to accommodate lattice mismatch. The structure included n-type In_0.1_Ga_0.9_N, n-type Al_0.15_Ga_0.85_N, and n-type GaN layers for carrier injection and optical confinement. The active region consisted of 26 In_0.2_Ga_0.8_N/In_0.05_Ga_0.95_N MQWs, followed by a p-type Al_0.2_Ga_0.8_N EBL and p-type GaN layers for hole injection and contacts (see Figure 6). Lasing was achieved at a relatively high operating voltage (~30 V), which prevented CW operation.

Although the initial device operated for only about one second, the threshold voltage was reduced to 8 V. By the end of the year, further optimization extended device lifetime to 27 h, lowered the voltage to 5.5 V, and maintained a threshold current density of ~3.6 kA/cm^2^. Subsequent design improvements led to the introduction of ridge geometry later that year, reducing the voltage to 20 V, the threshold current density to 3 kA/cm^2^, and the number of QWs to five. Continued progress in 1997 increased the lifetime to approximately 300 h.

In general, blue LDs exhibit higher EQEs than blue LEDs. Modern blue LDs typically achieve EQEs of ~20–45%, compared to peak values of ~80–90% for blue LEDs. This difference arises from the greater sensitivity of LDs to crystal defects, stronger Auger recombination at the high current densities required for lasing, carrier leakage from quantum wells, optical losses in waveguides and cavity mirrors, and the influence of polarization-induced electric fields in c-plane InGaN structures. Although LDs operate with smaller emission areas and therefore at much higher current densities, these conditions do not improve peak EQE; instead, they contribute to additional efficiency losses.

Following the successful demonstration of InGaN-based blue–violet LEDs and LDs, research rapidly expanded toward shorter emission wavelengths, based on AlGaN/AlGaN QWs, extending nitride emission into the UV spectral region. UV emitters are commonly classified as UVA (400–315 nm), UVB (315–280 nm), or UVC (280–200 nm), with their applications largely determined by photon energy and the corresponding interaction mechanisms with biological and chemical systems.

### 4.2. UVA (400–315 nm)

UVA emitters are widely used in photopolymerization and fluorescence excitation, with applications ranging from UV curing and 3D printing to microscopy, diagnostics, insect traps, and phosphor pumping for white light. Their lower photon energy and deeper penetration make them suitable for photochemical processes, with reduced biological risk compared to shorter UV wavelengths.

#### 4.2.1. LEDs

The first UV LED, emitting at 375 nm, was reported by Akasaki et al. [54] in 1993, with an EQE of 1.5%. It was grown using MOVPE and hydride vapor phase epitaxy (HVPE) on a highly mismatched substrate. This approach enabled the growth of high-quality nitride films on substrates such as α-Al_2_O_3_. Next, Mukai et al. [55] in 1998 achieved 7.5% EQE at 371 nm using the InGaN/AlGaN double heterostructure with an In composition of nearly zero. The undoped GaN layer acts as a buffer to reduce lattice mismatch, a diffusion barrier, and a stress-relief layer, thereby improving device performance and reliability. Progress continued, with Muramoto et al. [56] demonstrating EQEs up to 60% at 405 nm due to advances in crystal growth, chip processing, and packaging technologies. Later work by Oh et al. [57] showed that optimized AlN nucleation layers reduce defects and enhance EQE. The EQE at 370 nm was 43.7% for in situ AlGaN and 48.2% for ex situ AlN.

Designing efficient UV LEDs is challenging due to AlGaN material defects, low hole concentration, poor p-type contacts, limited light extraction, polarization-induced losses, and thermal issues. To address these, the research focuses on improving crystal quality and optimizing device architectures, including the use of patterned substrates and distributed Bragg reflectors (DBRs) [58]. For example, Lu et al. [59] demonstrated a 365 nm UVA LED incorporating nanoporous AlGaN DBRs with a reflectance of 93.5%. This design increased the EQE by ~54% and the output power by ~66% without degrading electrical performance, highlighting the strong potential of DBRs for enhancing light extraction in LEDs. However, despite their effectiveness, the application of DBRs in resonant-cavity nitride laser structures remains relatively limited.

In 2020, Li et al. [60] demonstrated high-efficiency 395 nm UVA LEDs grown on Si substrates using InGaN/GaN/AlGaN/GaN MQWs. A GaN interlayer barrier improved carrier concentration and overlap by tuning band structure and polarization effects, while also enhancing crystal quality through a two-step growth process. The resulting devices achieved 60% EQE and 659 mW output power at 350 mA injection current. 

#### 4.2.2. LDs

Early UVA LDs based on AlGaN and AlInGaN MQWs, typically grown on sapphire, exhibited high threshold current densities and were largely limited to pulsed operation. Their performance was constrained by high dislocation densities, inefficient p-type doping in Al-rich materials, and strong internal electric fields.

A review by Yang et al. [61] outlines key strategies for improving GaN-based UVA LDs (320–400 nm), including high-quality crack-free AlGaN templates, optimized active regions and waveguides, and improved thermal management. In 2003, Masui et al. [62] demonstrated CW operation at 365 nm with a 50 mA threshold current and ~2000 h lifetime. Later, Yoshida et al. [63,64] achieved AlGaN MQW LD emission at 342.3 and 336.0 nm, demonstrating the potential of AlGaN-based LDs for shorter-wavelength UV operation.

**Figure 7:**
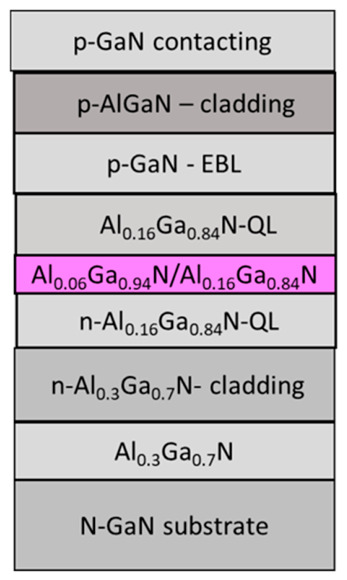
The schematic of the UVA LD epitaxial structure fabricated by Taketomi et al. [65].

Taketomi et al. [65] demonstrated high-peak-power operation of an AlGaN-based UVA LD emitting at 338.6 nm. The device was fabricated on a bulk GaN(0001) substrate, using a crack-free Al_0.3_Ga_0.7_N underlying layer grown by epitaxial lateral overgrowth (ELO). A broad-area, vertically conductive structure was employed to enhance output power. The device exhibited a threshold current density of 38.9 kA/cm^2^ and a differential EQE of 8.5%, with a characteristic temperature of 119 K and a wavelength temperature coefficient of 0.033 nm·K^−1^. A peak output power exceeding 1 W was achieved under pulsed operation at RT, representing one of the highest reported values until 2022. The schematic of their epitaxial structure is shown in Figure 7.

Zhao et al. [66] demonstrated CW GaN-based UV laser diodes in 2017, achieving emission at 392.9 and 381.9 nm with output powers of 80 mW and 14 mW, respectively. Yang et al. [67,68,69,70] showed that waveguide thickness plays a critical role in UV LD performance: thinner layers reduce carrier loss and threshold current, whereas excessively thin designs increase optical leakage. Optimized structures enabled emission down to ~358 nm and CW output powers up to 3.8 W at ~386 nm with wall-plug efficiencies approaching 20%.

Commercially, the Nichia Corporation has developed GaN-based LDs operating in the 375–380 nm range with stable CW operation, high output power, and a long lifetime, for applications such as optical storage and biomedical sensing [71].

### 4.3. UVB (315–280 nm)

UVB emitters are mainly applied in medical phototherapy, vitamin D synthesis, dermatological treatment, and biological and environmental research. UVB radiation induces controlled photochemical and biological responses, enabling therapeutic applications while avoiding the extreme DNA damage efficiency characteristic of UVC radiation.

In the early 2000s, fabricating UV LEDs with wavelengths below 300 nm was highly challenging due to the need for wide bandgap materials, which were scarce, difficult to process, and prone to absorption and scattering losses resulting in low efficiency.

#### 4.3.1. LEDs

The first sub-300 nm UVB LEDs were developed in 2000 by Nitride Semiconductor Co. in collaboration with the University of Tokushima (see the review by Muramoto et al. [56]).

In 2009, Hirayama et al. [72] achieved 1.2% EQE at 282 nm using LEDs grown on low-threading-dislocation AlN templates and fabricated by an NH_3_ pulse–flow multilayer technique. The devices included 222–273 Al_x_Ga_1−x_N/Al_y_Ga_1−y_N-based LEDs, with x = 0.83–0.86 and y = 0.89–0.67, and >280 nm InAlGaN-based LEDs with high Al content. In 2010, Shur et al. [73] demonstrated a GaN-based UV LED with an efficiency of 0.01% at wavelengths below 300 nm and Fujioka et al. [74] improved the efficiency of 280 nm UV LEDs to approximately 2.78%. This was achieved by employing high-crystal-quality AlN templates with optimized epitaxial structures.

Significant progress in UVB LED performance was reported by Khan et al. [75] in 2020, who demonstrated AlGaN-based LEDs emitting at 310 and 294 nm through optimized carrier transport and MQW design. The 310 nm device achieved 29 mW output power and 4.7% EQE, while the 294 nm LED with increased Al content in the undoped AlGaN layer reached 32 mW and 6.5% EQE under pulsed operation at RT. Further improvements were reported by the same authors in 2025 [76], with EQE reaching 9.6% for 304 nm AlGaN-based UVB LEDs. The enhancement resulted from improved light extraction using nano-patterned sapphire substrates, photonic crystals, and Al reflectors.

In 2023, Wang et al. [77] investigated challenges in growing highly conductive n-AlGaN layers for germicidal UV (GUV) LEDs. They showed that thick, heavily Si-doped n-AlGaN suffers from surface degradation caused by the Si antisurfactant effect, resulting in dislocation inclination, strain relaxation, cracking above 400 nm thickness, and unintended Ga incorporation. By optimizing the n-AlGaN conductivity and using thinner conductive layers with a smoothing SL, they demonstrated 285 nm UV LEDs with a low forward voltage of 4.2 V and a peak EQE of 10.6%.

At the same time, several groups reported UV-emitting micro- and nano-LEDs suitable for high-performance displays due to their high efficiency, brightness, and stability. However, in these and similar studies, EQE decreases with device size and remains lower than in conventional MQW structures, dropping from ~10% for ~10 μm devices to ~2–3% at ~1 μm [78]. Zhao et al. [79] fabricated AlGaN-based micro-ring LEDs and showed that optimized ring geometries improve optical emission and light extraction efficiency compared with conventional micro-circular LEDs. Their best device, emitting at ~280 nm, achieved a light output power density of 53.36 W/cm^2^ at 650 A/cm^2^ and an EQE of 6.17%, demonstrating the potential of ring-shaped micro-LEDs for enhanced UV emission.

#### 4.3.2. LDs

The first electrically pumped AlGaN-based thin-film laser operating below 300 nm was demonstrated by Zhang et al. [80] in 2019 at 271.8 nm, followed by RT pulsed UVB lasers emitting at 298 nm (Sato et al. [81]). Subsequent improvements in 298 nm laser performance were reported by Omori et al. [82], while electrically pumped 310 nm UVB lasers were demonstrated in 2021 by Hjort et al. [83].

### 4.4. UVC (280-200)

UVC nitride emitters are primarily used for disinfection and sterilization in medical equipment, wastewater treatment, air purification, and food and packaging sterilization. Their effectiveness arises from the high photon energy, which directly damages microbial nucleic acids, leading to efficient inactivation of bacteria, viruses, and other pathogens.

#### 4.4.1. LEDs

UV optoelectronic devices are typically fabricated using AlGaN-based heterostructures. Early AlGaN-based UVC LEDs exhibited high threshold current densities and operating voltages due to poor material quality, weak optical confinement, high internal losses, severe self-heating, and limited device lifetime.

In 2012, Shatalov et al. [84] enhanced the performance of AlGaN-based UVC LEDs grown on sapphire substrates by optimizing chip encapsulation. As a result, they achieved an EQE of 10.4% at 20 mA CW operation, with output power up to 9.3 mW at 278 nm. Hirayama et al. [85] (2014) reported AlGaN-based DUV LEDs operating from 222 to 351 nm. Improved crystal growth, low-TDD AlN buffers, MQB structures, and enhanced light extraction enabled a maximum EQE of 7% at 279 nm.

Bryan et al. [86] studied Al_0.55_Ga_0.45_N/Al_0.85_Ga_0.15_N based UVC MQWs grown on bulk AlN substrates and achieved a record IQE of ~80% at 258 nm under high V/III growth conditions, attributed to reduced non-radiative recombination and improved MQW quality. To improve EQE, Takano et al. [87] employed a transparent Al_0.65_Ga_0.35_N:Mg contact layer, a Rh mirror electrode, growth on an AlN template over patterned sapphire, and resin encapsulation. Significantly enhanced light extraction resulted in a maximum EQE of 20.3% at 275 nm. Maeda et al. [88] achieved 9% EQE at 279 nm using transparent contact layers and reflective Ni/Al p-electrodes. An optimal Ni thickness of ~0.9 nm provided the best balance between reflectivity and efficiency.

In 2020, Pandey et al. [89] investigated LEDs operating near 265 nm and achieved an EQE of 11% using an AlGaN/GaN/AlGaN tunnel-junction structure. A thin GaN layer inserted between p^+^- and n^+^-AlGaN layers reduced the tunneling barrier and improved carrier injection. In 2021, Zheng et al. [90] reported 5.19% EQE at 273 nm using a double-layer nano-patterned array that improved light extraction by diffraction and reduced absorption.

Matsukura et al. [91] studied how the optical thickness of p-layers affects light output in AlGaN-based deep-UV LEDs with transparent high-Al-content p-AlGaN cladding, a thin p-GaN contact layer, and a reflective p-electrode. Maximum output was obtained when the total p-layer optical thickness was ~0.66λ. At 275 nm, optimized devices achieved 385 mW output power at 1500 mA, a peak EQE of 15.7%, and a maximum wall-plug efficiency of 15.3% at 10 mA. The LEDs retained 85% of initial output after 1000 h, showing that optimizing p-layer optical thickness is crucial for improving light extraction.

Liu et al. [92] (2024) improved 270-nm AlGaN-based DUV LEDs, using optimized MQWs, a reflective Al mirror, a low-loss tunneling junction, and a SiO_2_ insertion layer. The devices achieved 140.1 mW output power at 850 mA and an EQE of ~6.9%, while reducing QCSE, p-layer absorption, and poor TM-polarized light extraction.

Many other studies in the UVC spectral region have been carried out based on QDs. Among these, Yang et al. [93] demonstrated MOVPE-grown GaN/AlN QD LEDs emitting at 309 nm with stable emission up to 300 K and IQE of ~62% at RT. Brault et al. [94] reported MBE-grown AlᵧGa_1−_ᵧN QDs with tunable emission from 332 to 276 nm and low-temperature IQE up to 66%. Stachurski et al. [95] confirmed strong RT emission and exciton confinement in GaN/AlN QDs, while Zaiter et al. [96] achieved strong UV-C emission at 275–280 nm from MBE-grown Al_0.3_Ga_0.7_N QDs on ultrathin h-BN/sapphire templates.

#### 4.4.2. LDs

Nitride emitters in the UVC range are currently limited mainly to LEDs, while LDs remain largely at the research stage due to significant material and device challenges.

Major advances in AlGaN-based LDs operating in the 200–280 nm range were reported by Zhang et al. [80,97,98] and Kushimoto et al. [99]. Key developments included high-quality AlGaN growth on single-crystal AlN substrates and improved p-type conductivity using distributed polarization doping. These advances enabled RT pulsed lasing and later CW operation at 5 °C through reduced dislocation density and optimized device design. CW lasing at 274.8 nm was achieved above 110 mA, with a threshold current density of 3.7 kA cm^−2^ and a low threshold voltage of 9.6 V, using a double-sided n-electrode configuration.

Table 1 summarizes the performance of representative blue, violet, and UV LEDs and LDs discussed in this work, including emitter type and key characteristics, emission wavelength, EQE, and corresponding references.

**Figure 8:**
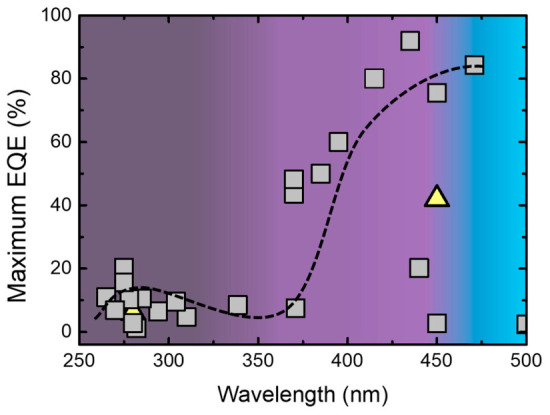
EQE as a function of emission wavelength for UV, violet and blue emitters. The dashed line is a guide for the eye. Triangle refers to micro-LED [79].

Figure 8 shows the dependence of EQE on emission wavelength for the nitride light emitters listed in Table 1, illustrating the observed EQE trend. Figure 8 shows that InGaN-based blue LEDs achieve very high EQE (>70% at 440–450 nm) despite the high dislocation densities typical of heteroepitaxial III-nitride growth. Unlike conventional III-V LEDs, InGaN devices remain efficient even at dislocation densities near ~10^10^ cm^−2^ due to the strong ionic bonding in III-nitrides, which suppresses non-radiative recombination at dislocations [100]. Further improvements arise from carrier localization effects in InGaN, optimized structures, enhanced carrier injection, and improved light extraction.

The lower EQE below 400 nm in the UVA region mainly results from the transition from InGaN/GaN to AlGaN/GaN material systems. Compared with InGaN, AlGaN exhibits weaker carrier and exciton localization, leading to enhanced non-radiative recombination. Poorer crystal quality, strain-related defects, and reduced optical confinement and light extraction further limit the quantum efficiency.

In the UVB region, EQE increases due to stronger carrier confinement in higher-Al-content AlGaN QWs, which suppresses carrier leakage and enhances radiative recombination. However, below ~280 nm (UVC range), the EQE decreases again because very high Al-content AlGaN suffers from poor crystal quality, inefficient p-type doping, and high defect densities. In addition, the emitted light becomes predominantly transverse magnetic (TM) polarized, causing more light to propagate parallel to the layers rather than out of the device, thereby reducing light-extraction efficiency.

**Figure 9:**
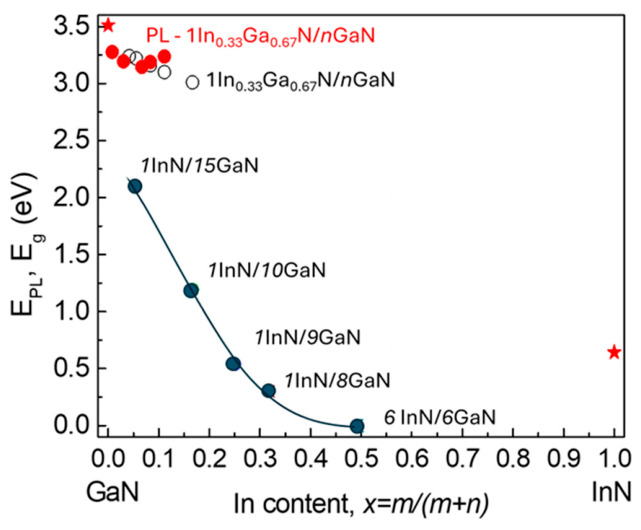
Calculated band gaps, *E_g_*, of *m*InN/*n*GaN and 1In_0.33_Ga_0.67_N/*n*GaN SLs as functions of effective In content, *x* = *m*/(*m* + *n*), in comparison with the experimental data (red dots and stars). Based on Figure 1 Ref. [105] and on Figure 3 Ref. [107].

A similar EQE–wavelength dependence for blue to UV emitters, which was based on a broader dataset, was reported by Kneissl et al. [101]. A comparison of Figure 5 and Figure 9 in their work reveals the same overall trend. Efficiency improvement strategies in LEDs, highlighting progress driven by optimized device design, composition, and growth techniques, were reviewed in 2024, by Bhattarai et al. [102].

## 5. Nitride Light Emitters: From Green to Red (500–750 nm)

Red and green emitters are both essential for modern optoelectronic technologies, particularly in full-color displays, solid-state lighting, and laser-based systems. Along with blue emitters, they form the RGB platform necessary for a wide color gamut, high color fidelity, and brightness in LEDs, LDs, and emerging micro-LED displays. Green emission is especially important because the human eye is most sensitive in the green spectral region, making it critical for luminous efficiency, while red emission enables accurate color balance and extends functionality into sensing, communication, and biomedical applications.

### 5.1. Problems with Indium Content in InGaN QWs

Achieving high efficiency in the green-to-red spectral range remains challenging for III-nitride materials because longer wavelengths require higher indium incorporation in InGaN QWs. This increases strain, defects, compositional inhomogeneity, and QCSE, reducing radiative recombination efficiency. Lower growth temperatures and lattice mismatch further degrade material quality. These effects cause a strong efficiency drop toward the red region and are even more critical in LDs, where high material quality and optical gain are required, limiting the performance of green LDs and preventing practical red nitride-based LDs.

Yoshikawa [103] proposed an early strategy to improve long-wavelength III-nitride emitters by using binary InN/GaN SLs with ultrathin InN layers instead of high-In-content In_x_Ga_1−x_N alloys placed in wide QWs. Since In-rich InGaN alloys tend to undergo phase separation and compositional fluctuations, the SL approach aimed to achieve better structural uniformity and more precise band-gap control while avoiding the limitations associated with high In incorporation.

Gorczyca et al. [104,105] predicted from ab initio calculations that the band gap in mInN/nGaN superlattices (SLs) decreases systematically with increasing InN and GaN layer thicknesses. A 1InN/15GaN SL was calculated to have a band gap of ~2.2 eV (~570 nm), in good agreement with the value reported by Miao et al. [106] (~2.17 eV for a 1InN/23GaN structure using multiband *k·p* modeling), suggesting the possibility of emission deep in the green spectral range. Extending these calculations over a broad range of SL periods revealed a continuous reduction of the band gap with increasing layer thickness. For sufficiently thick structures (*m*, *n* ≳ 6 monolayers), the band gap was predicted to close completely, indicating a polarization-induced semiconductor-to-metal transition driven by strong internal electric fields in the WZ structure. The calculated trends are illustrated in Figure 9.

**Figure 10:**
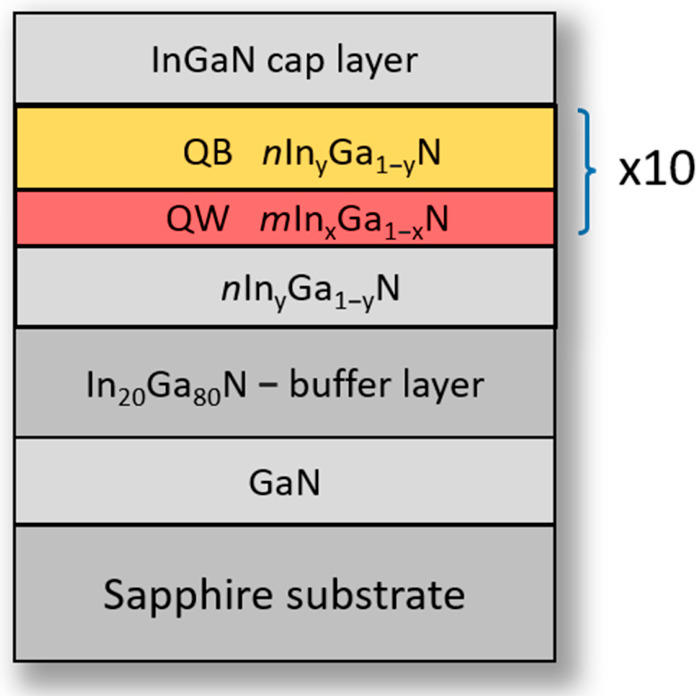
Schematic diagram of the SLs investigated in Ref. [112].

However, fabrication of ideal InN/GaN SLs with 1–2 MLs of pure InN proved extremely difficult. Suski et al. [107] showed by high-resolution transmission electron microscopy (HRTEM) studies that nominal 1InN/GaN SLs actually contained In_x_Ga_1−x_N layers with x ≈ 0.33 instead of pure InN. The measured PL energies agreed well with calculations for 1In_0.33_Ga_0.67_N/nGaN SLs (see Figure 10), highlighting both the promise of binary SLs for band-gap engineering and the practical limitations imposed by growth kinetics, interdiffusion, strain, and limited In incorporation in pseudomorphic InGaN/GaN QSs. Furthermore, it was recognized that limited indium incorporation—typically not exceeding approximately 30–33%—is a general characteristic of all pseudomorphically grown InGaN/GaN QSs.

Duff al. [108] and Lymperakis et al. [109] showed theoretically that strong lattice-mismatch strain makes pseudomorphic InN growth on GaN energetically unfavorable, limiting stable In incorporation in coherent InGaN layers to about 25%. Their results also suggested that higher In incorporation could be achieved using In_0.25_Ga_0.75_N templates with reduced lattice mismatch, motivating the development of compositionally graded structures to alleviate strain effects. A few subsequent studies experimentally followed these suggestions [104,105,106].

Dussaigne et al. [110] used a relaxed InGaN pseudo-substrate to increase In incorporation and extend emission toward longer wavelengths. Reduced lattice mismatch lowered strain and compositional pulling effects, leading to strong PL redshifts compared with GaN substrates and enabling emission in the amber (~594 nm) and red (~624 nm) spectral regions.

Siekacz et al. [111] studied InGaN/GaN short-period SLs grown by plasma-assisted molecular beam epitaxy (PAMBE) on GaN and partially relaxed In_0.2_Ga_0.8_N buffer layers. PL measurements showed a systematic redshift of the emission peak with an increasing in-plane lattice constant, from 379 nm for GaN substrates to 419 nm for In_0.2_Ga_0.8_N buffers. Staszczak et al. [112] performed similar studies on InGaN/GaN SLs grown by MOVPE on InGaN buffer layers with 17% and 20% In. A schematic diagram of such an SL structure is shown in Figure 10. Increasing the In content in the buffer to 20% significantly reduced the effective band gap, producing a redshift of about 0.72 eV (~167 nm) and extending the emission wavelength to ~590 nm in the amber spectral range.

The above experiments demonstrate that growth on relaxed InGaN buffers reduces lattice mismatch, enabling higher In incorporation in the InGaN QWs of the SL. Relaxed or partially relaxed InGaN buffer layers were subsequently adopted in various nitride light-emitting structures to facilitate longer-wavelength emission.

### 5.2. Green (500–590 nm)

#### 5.2.1. LEDs

The first blue–green LEDs based on III-nitride semiconductors were demonstrated in the mid-1990s. In 1994, Shuji Nakamura and co-workers [44] reported blue–green LEDs exhibiting a luminous intensity of 2 cd. This performance was achieved by increasing the In composition in the InGaN active layer to 23% and by co-doping the layer with Zn and Si to optimize carrier concentration and radiative recombination efficiency. In a subsequent report published in 1995, Shuji Nakamura et al. [113] demonstrated super-bright green LEDs employing a p-AlGaN/InGaN/n-GaN heterostructure grown by MOCVD on sapphire substrates. These devices exhibited a luminous intensity of 12 cd and EQE 6.3% at a forward current of 20 mA, the output optical power was 3 mW and the EL peak wavelength was 520 nm.

After the initial demonstrations of green InGaN LEDs, major challenges remained in achieving higher efficiency and longer wavelengths. Further progress is fundamentally limited by strong polarization-induced electric fields that reduce radiative recombination. A common approach to suppress these fields is growth along semipolar or nonpolar directions, but practical implementation is hindered by growth complexity, limited In incorporation, and the lack of high-quality substrates. Other explored strategies included improving crystal quality, using AlGaN interlayers, utilizing pre-strained templates, and employing QDs or nanowires.

In 2018 Li et al. [114] demonstrated InGaN-based green LEDs grown on c-plane patterned sapphire substrates using MOCVD. The 527 nm green LEDs exhibited a high EQE of 53.3%, WPE of 54.1%, and a peak luminous efficacy of 329 lm/W. Zhou et al. [115] systematically studied mechanisms for efficient green LEDs. Using cathodoluminescence and Raman measurements, they showed that V-pit-induced potential barriers in InGaN/GaN structures suppress non-radiative recombination at threading dislocations, significantly improving IQE. The barrier height depends on V-pit diameter, influencing efficiency, forward voltage, and droop. Optimized V-pit structures enabled 525 nm green LEDs with an EQE of 42%.

Leem et al. [12] investigated the low efficiency of green LEDs using microscopic PL analysis of GaN-based InGaN/GaN MQWs. They found that In-enriched clusters, previously considered efficient emitters, actually localize excessive carriers and promote non-radiative recombination. Their formation through metastable phase separation also degrades the surrounding crystal quality, effectively reducing the active volume of the LED.

**Figure 11:**
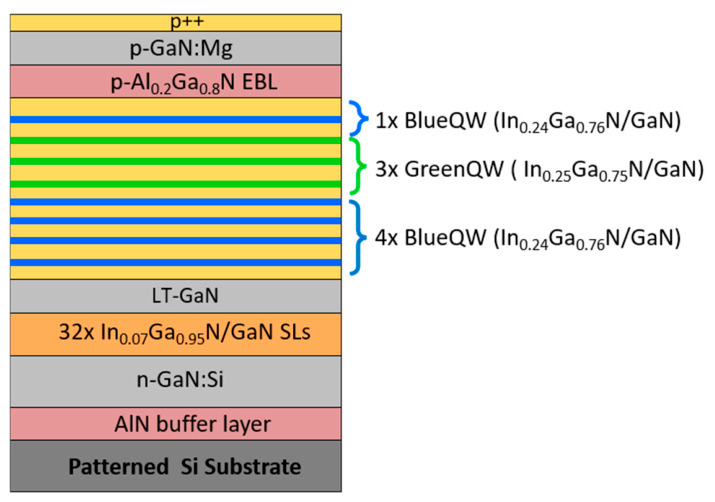
Schematic of the epitaxial layer structure of the InGaN/GaN green LED described in the text. Based on Figure 1 of Ref. [116].

Lv et al. [116] reported 525 nm green LEDs with EQE up to 55.6%, using an optimized InGaN/GaN MQW structure grown on PSS by MOCVD. The device structure included an AlN buffer layer, a 3.3 µm thick n-type GaN layer, and 32 periods of InGaN/GaN SL for strain relief. The active region consisted of a hybrid MQW design comprising four In_0.24_Ga_0.76_N/GaN layers, followed by three interior In_0.25_Ga_0.75_N/GaN layers with the same QW thickness of 3 nm and QBs of varying thicknesses, and finally one In_0.24_Ga_0.76_N/GaN layer on the top. Performance improvement was achieved by optimizing the thicknesses of the three inner QBs. The structure is illustrated in Figure 11.

Guo et al. [117] studied AlGaN interlayers in GaN-based green LEDs on silicon. The interlayers improve luminous efficiency by enhancing carrier distribution, suppressing dislocations, and reducing InGaN phase separation via increased compressive stress. They also strengthen V-pit effects. However, under electrical stress, they promote defect formation, causing light degradation at low current. The 525 nm LEDs achieved a peak EQE of 50%.

Choi et al. [51] investigated efficiency droop in blue and green GaN-based LEDs and LDs using epitaxial structures with varying indium content. For green emission, the In content in the QWs was 25%, while the QBs contained 2% indium, corresponding to an emission wavelength of 520 nm. The reported EQE was 78.8%.

Numerous other studies have explored strategies to improve green LED performance. Liu et al. [118] introduced V-pit-embedded InGaN/GaN SLs that suppressed non-radiative recombination and enhanced carrier injection, increasing EQE by ~30% at 20 mA. Hu et al. [119] demonstrated high-efficiency green LEDs using an InGaN/GaN quasi-superlattice interlayer and Al-doped ITO, achieving a luminous efficacy of 264.7 lm/W at 20 mA and 537.2 nm emission. Zhou et al. [120] improved GaN-based green LEDs by employing InGaN QWs with graded In composition, resulting in higher light output and reduced efficiency droop.

Low-dimensional structures, including micro- and nanostructures, QDs, and nanowires, have emerged as alternative active-region designs for overcoming limitations in indium incorporation. In contrast to planar MQWs, QDs provide strong three-dimensional carrier confinement, reduced sensitivity to threading dislocations, and partial strain relaxation, enhancing radiative recombination efficiency—particularly at longer wavelengths. Their nanoscale dimensions also allow substantially higher local indium concentrations (~40–50%), enabling emission in spectral regions difficult to achieve with conventional QWs.

However, reducing device dimensions increases the surface-to-volume ratio, allowing a larger fraction of carriers to reach surface states and defects, which can enhance non-radiative recombination and reduce EQE. Despite this limitation, μLEDs and nano-LEDs can benefit from improved light extraction and reduced efficiency droop, making them particularly attractive for green InGaN emitters.

Smith et al. [78] compared the EQE trends of blue and green InGaN micro-LEDs and found that green-wavelength devices are less susceptible to efficiency degradation with decreasing device size. This behavior can be explained by carrier localization effects. Higher In content, required for green emission, introduces stronger potential fluctuations in the InGaN alloy. These fluctuations localize electrons and holes in small regions, limiting their diffusion to the device surface and thereby reducing surface-related non-radiative recombination.

The potential of QDs for extending emission into the yellow–green regime was comprehensively reviewed by Weng et al. [121], who summarized fabrication methods for III-nitride QDs and their application in QD-based LEDs, lasers, infrared photodetectors, and intermediate-band solar cells. Notably, electrically injected InGaN/GaN QD lasers emitting at λ ≈ 524 nm have been demonstrated, highlighting the viability of QDs for long-wavelength nitride emitters. In the same spectral range, Lv et al. [122] demonstrated that multilayer InGaN/GaN QD structures grown by MOVPE constitute a promising active region for yellow–green LEDs. After optimization of the growth conditions, a 10-layer QD LED was realized, with transmission electron microscopy (TEM) confirming the formation of uniform, vertically aligned QDs. EL measurements revealed a pronounced blueshift of the emission wavelength-from 537 nm to 574 nm as the injection current increased from 5 to 50 mA—attributed to carrier-induced screening of internal electric fields and state-filling effects.

**Figure 12:**
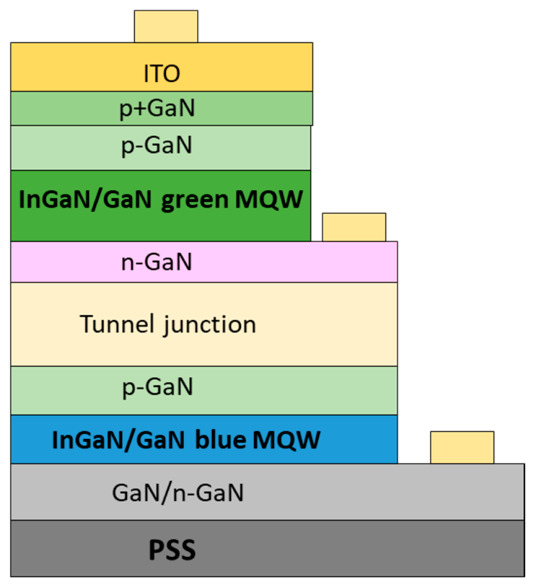
The epitaxial layer structure of the cascaded blue and green μLEDs. Based on Figure 2, Ref. [49].

As mentioned already in the previous section, Li et al. [49] demonstrated cascaded blue/green μLEDs. The green μLEDs (40 × 40 μm^2^) exhibited a forward voltage of 3.1 V at 20 A/cm^2^ and EQE of 14%. The emission peak was 518 nm. The epitaxial structure is shown in Figure 12.

Liu et al. [123] in 2022 demonstrated N-polar InGaN nanowire LEDs grown by PAMBE with nearly size-independent efficiency scaling. EQE values reached approximately 11% for devices as small as 750 nm in the lateral dimension without packaging. The improved performance is attributed to reduced defect densities, suppressed non-radiative recombination, and better carrier confinement in nanowire geometries. Additionally, these studies examine the influences of carrier leakage and Auger recombination, providing further insight into the limiting mechanisms at high injection levels.

**Figure 13:**
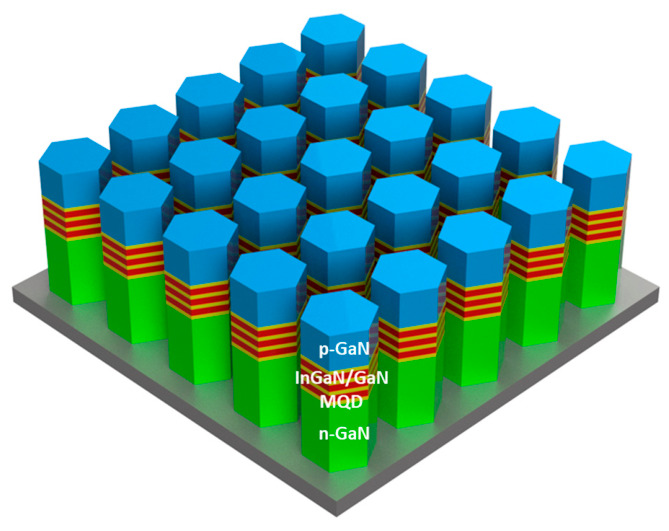
Schematic of the N-polar InGaN/GaN nanowire excitonic LED heterostructure containing multiple quantum disks. Based on Figure 1 of Ref. [124].

In 2023, Pandey et al. [124] demonstrated submicron-scale green-emitting LEDs with an EQE and wall-plug efficiency of 25.2% and 20.7%, respectively, and emission wavelengths of about 490–515 nm. They identified several critical factors for achieving excitonic micro-LEDs, including the epitaxial growth of nanostructures to enable strain relaxation, the use of semipolar planes to minimize polarization effects, and nanoscale quantum confinement to enhance electron-hole wavefunction overlap. Schematic of the N-polar InGaN/GaN nanowire excitonic LED with multiple quantum disks is shown in Figure 13.

Smith et al. [125] (2024) identified two size-dependent effects that can improve the efficiency of InGaN μLEDs. First, reducing the device size increases light directionality and extraction efficiency. Second, higher indium content suppresses surface recombination. Together, these effects help counteract the efficiency loss typically observed in smaller μLEDs, with the improvement becoming stronger as the indium content increases from blue to red emitters. As the μLED diameter decreased from 50 to 1 μm, the EQE of 500 nm devices dropped slightly from 16.5% to 14%, whereas the EQE of 600 nm devices increased significantly from 2.7% to 7.1%.

Despite these advances, challenges related to QD size uniformity, emission wavelength homogeneity, and large-area scalability continue to limit widespread industrial adoption. Nevertheless, QDs remain a promising next-generation active-region concept.

Device scaling effects remain a critical challenge for μLEDs applications. Studies investigating size-dependent behavior show that as the device dimensions shrink, emission characteristics become increasingly sensitive to current density and strain relaxation.

Although the green gap is gradually narrowing, it has not yet been fully overcome in commercial LED production. On the other hand, the most significant progress has been achieved through advances in epitaxial growth and optimization of QW and waveguide designs in conventional c-plane multiple MQW structures.

Current research focuses on new materials, such h-BN, nitride/oxide heterostructures, and advanced growth techniques, including PAMBE. Recent PAMBE-grown green emitters exhibit reduced efficiency droop, suggesting that improved indium incorporation and lower defect densities may help mitigate the green gap. These findings indicate that the green gap is likely not a fundamental physical limitation, but rather a consequence of challenges associated with material growth and device design.

#### 5.2.2. LDs

For green LDs, Choi et al. [51] (2024) reported an EQE of ~23.6% at an emission wavelength of 500 nm. Numerous other studies have focused on improving the performance of green laser diodes. For example, Yang et al. [126] showed that increasing the In content in (In)GaN barriers up to ~5% improves optical confinement and reduces the threshold current in green LDs, whereas higher In contents degrade performance due to carrier leakage. This effect can be mitigated by reducing the thickness of the last barrier, highlighting the advantages of asymmetric MQW designs. In addition, Hu et al. [127] demonstrated a hybrid green LD with an ITO cladding layer, reducing the threshold current density from ~5 to 1.6 kA/cm^2^, increasing slope efficiency, and enabling output powers up to 400 mW.

Further wavelength extension and improvements in efficiency remain fundamentally constrained by strong polarization-induced electric fields, which reduce radiative recombination rates. A widely adopted approach to mitigating or completely eliminating these fields is the growth of QW structures and related emitters along semipolar or nonpolar crystallographic orientations, where polarization effects are significantly suppressed. The fabrication of nonpolar and semipolar InGaN-based green LDs with substantially reduced internal electric fields has already been demonstrated [128,129,130]. Reports have shown lasing in the 505–526 nm spectral range, accompanied by improved device characteristics such as lower threshold current densities, enhanced electron-hole wavefunction overlap, higher active-region material quality, improved emission uniformity, and reduced polarization-induced blueshift. Despite their clear physical advantages, the performance and efficiency of nonpolar and semipolar LDs have so far remained below initial expectations, mainly due to material and structural limitations similar to those encountered in LEDs. Green LDs produced on an industrial scale are typically grown as polar structures and operate in the 510–530 nm range, achieving EQE values of ~20–25% at moderate injection currents. These devices are widely used in display and projection systems.

### 5.3. Yellow–Amber (590–620)

Yellow and amber emission are particularly important for applications such as micro-displays, automotive lighting, and visible-light communication. Yellow emission also plays a key role in warm-white lighting and enables improved color mixing in RGB or multi-primary display systems. Yet this emission remains challenging due to efficiency limitations in high-indium-content InGaN.

In 2016 Iida et al. [131] demonstrated the effectiveness of a MQWs structure containing blue and orange light-emitting QWs. They achieved relatively narrow emission at 620 nm with full width at half maximum (FWHM) of 51 nm. However, the efficiency of such devices remains limited, with reported EQE values around 0.6%, indicating that further optimization is needed. Nevertheless, these designs provide valuable insight into spectral engineering approaches for multi-color emission. Yu et al. [132] studied high-In InGaN QDs grown by MOVPE, achieving 613 nm emission. μLEDs (1–20 µm) based on these QDs showed up to 4.9% EQE. By introducing pre-strained MQW layers, emission was extended to 638 nm, though with reduced efficiency. These results highlight the potential of InGaN QDs for red μLED applications, despite current efficiency limitations.

Horng et al. [133] observed that smaller InGaN red micro-LEDs exhibit pronounced blue shifts in emission wavelength with increasing injection current, with shifts from ~617 nm to ~577 nm in 10 × 10 μm^2^ devices, with a maximum EQE of 5%. This phenomenon is attributed to a combination of stress relaxation and high carrier injection levels. Interestingly, the output power density remains relatively constant across different device sizes at the same current density, suggesting that sidewall passivation can effectively mitigate surface recombination effects. These findings contrast with the behavior of AlGaInP-based devices and highlight unique advantages of InGaN material systems. In addition to this, one should remember that profits from using one semiconductor family simplify (i) epitaxial growth (same substrates, like sapphire or GaN), (ii) multicolor device fabrication, and (iii) integration into monolithic RGB (red, green, and blue), i.e., all colors on one chip.

Ewing et al. [134] in 2023 reported V-defect-engineered LEDs on (0001) PSS, achieving EQE of 6.5% at 600 nm. The enhancement was attributed to improved lateral carrier injection via V-defects introduced by an InGaN/GaN SL. However, despite the advantages of PSS, its lower threading dislocation density limits V-defect formation, and TEM analysis revealed additional defect states, including stacking fault-related clusters, which are particularly detrimental in red/amber devices due to their impacts on efficiency and potential leakage pathways. In the same year Li et al. [135] showed that QW engineering significantly enhances InGaN red μLED performance, achieving EQEs of 6.0% (80 × 80 μm^2^) and 4.5% (5 × 5 μm^2^), which was attributed to improved electron-hole wavefunction overlap. The epitaxial structure consists of a 3 μm unintentionally doped (UID) GaN layer, 3 μm Si-doped n-GaN, 30-period InGaN/GaN SLs, 6-period InGaN/AlGaN/GaN MQWs, a 20 nm AlGaN EBL, 120 nm Mg-doped p-GaN, and a 20 nm heavily Mg-doped p^+^-GaN cap.

### 5.4. Red (620–750)

Nitride red emitters are typically realized using nanostructures. In conventional InGaN/GaN QWs the practical indium content is typically limited to ~25–30%. In contrast, ultrathin QWs and nanoscale structures—particularly QDs—can accommodate significantly higher local indium concentrations (~40–50%) because their small dimensions enable partial strain relaxation. As a result, QDs and related nanostructures allow emission to extend into the red and near-infrared spectral ranges that are difficult to achieve in planar quantum wells. Compared with MQWs, QDs provide strong three-dimensional carrier confinement, reduced sensitivity to dislocations, and partial strain relaxation, thereby improving radiative efficiency in long-wavelength micro- and nano-LEDs. However, challenges related to size uniformity, wavelength homogeneity, and large-scale fabrication still limit their industrial adoption.

Red light emission in semiconductor devices is currently realized using two competing material systems: AlGaInP and InGaN. AlGaInP alloys enable highly efficient emission in the 600–650 nm range, benefiting from negligible QCSE and excellent lattice matching to GaAs substrates, which ensures low defect densities and high radiative recombination rates. However, their performance degrades significantly in micro-devices (<20 µm), primarily due to enhanced surface recombination, long carrier diffusion lengths, and etch-induced defects.

InGaN-based emitters, on the other hand, struggle with material issues at high indium content (strain, defects, and strong QCSE), making red emission harder to achieve. However, they perform better at smaller scales thanks to carrier localization, which limits non-radiative losses and reduces size dependence. As a result, InGaN-based red micro-LEDs are among the most promising options for long-wavelength visible emission, particularly where size effects limit the performance of traditional AlGaInP materials.

The key divergence between these material systems lies in their recombination mechanisms: diffusion-driven transport in AlGaInP versus localization-driven recombination in InGaN. As device dimensions shrink, the performance-limiting factor shifts from bulk material quality to carrier confinement. This transition explains the growing preference for InGaN in micro-LED applications, despite its intrinsic material challenges, particularly the difficulty of incorporating more than ~25–30% indium into InGaN QWs.

Red nitride-based emitters remain significantly more challenging to realize than their blue and green counterparts, although substantial progress has been made in recent years, particularly by the Nichia Corporation and other research groups. Key advances include

- Demonstration of true red InGaN LEDs (620–640 nm);
- EQEs exceeding 10–20%, with rapid improvements;
- Development of red InGaN micro-LEDs, critical for next-generation displays;
- Potential for monolithic RGB emitters using a single material system.

Despite significant progress in InGaN-based red LEDs, as reported by Lu et al. [136], as to extended emission wavelengths and improved quantum efficiency in nitride LEDs, efficient red-emitting nitride LDs have not been realized, mainly due to material limitations associated with high In content.

Recent progress in InGaN-based red micro-/nanoLEDs focuses on overcoming efficiency losses caused by high indium content, poor crystal quality, and strong polarization fields—issues intensified by device miniaturization. Current strategies include nanostructuring, strain engineering, doping optimization, and advanced device designs.

Huang et al. [137], in 2022, demonstrated that device-level engineering played a crucial role in improving performance. The integration of SL structures, atomic layer deposition passivation, and DBRs enhances both carrier confinement and light extraction efficiency. Using these techniques, red InGaN micro-LEDs have achieved EQE values exceeding 5% with reduced efficiency droop at high current densities. Moreover, fast carrier recombination dynamics were revealed, enabling modulation bandwidths of up to 271 MHz and data transmission rates of 350 Mbit/s, thereby demonstrating the potential of these devices for high-speed visible light communication.

One of the most promising approaches involves the use of nanowire-based device geometries, particularly N-polar InGaN nanowires, which inherently reduce threading dislocations and allow for improved strain relaxation. Pandey et al. [138], in 2022, demonstrated that dislocation-free N-polar InGaN/GaN nanowire LEDs incorporating an InGaN/GaN short-period SL beneath the active region enable enhanced indium incorporation and reduced QCSE, resulting in a red emission exceeding 620 nm with a peak EQE of 2.2% at submicrometer dimensions. This work highlights the importance of strain management at the nanoscale to enable longer wavelength generation and emission. In the subsequent year, Pandey et al. [139] demonstrated bottom-up fabricated nanowire LEDs with optimized Mg doping in the p-GaN region, significantly improving hole injection efficiency, and achieving EQE values up to ~8.3% for red emission beyond 630 nm. These findings emphasize the critical role of p-type doping optimization in overcoming carrier imbalance in high-indium-content systems.

Chen [140] (2023) reported high-performance InGaN red LEDs on sapphire grown by MOCVD using strain modulation based on grain coalescence in the composite buffer. A composite buffer layer increases the surface lattice constant of GaN, enhancing indium incorporation in the InGaN active layers. As a result, red mini-LEDs with a peak wavelength of 629 nm and an EQE of 7.4% were achieved. Using these devices, a 60 × 90 pixel full-color nitride mini-LED display was demonstrated, highlighting the potential of all-nitride high-resolution mini/micro-LED displays.

In parallel, substantial progress has been achieved by Lee et al. [141] in planar InGaN LED structures through strain and band structure engineering. The incorporation of GaN cap layers and AlGaN interlayers within InGaN QWs has been shown to effectively modulate the electronic band structure, inducing band bending and modifying electron wavefunction overlap. This approach enables a controlled red shift of emission wavelength to approximately 625 nm while maintaining high efficiency, with reported EQE values of 10.5% at a current density of 10 A/cm^2^. Such band engineering techniques are crucial for achieving longer wavelength emission without excessively increasing indium content, which would otherwise degrade material quality.

Another effective strain management strategy, by Xing et al. [142], involves the use of advanced template engineering. By growing GaN on hexagonal columnar structures formed on porous SiN_x_ masks and subsequently coalescing them into a continuous template, it is possible to significantly reduce internal stress and threading dislocation densities. This enables the realization of deep-red InGaN LEDs with emission wavelengths as long as 672 nm and EQE values of 9.1% at low current densities. After some improvements in the applied approach, Xing et al. [143] demonstrated LED structure with emission wavelength shifted to 682 nm and an EQE of 9.2%. These results demonstrate that careful control of the underlying template LED structure can directly influence the achievable emission wavelength and efficiency in InGaN systems.

In summary, the collective body of work reviewed here demonstrates significant progress toward overcoming the efficiency limitations of InGaN-based red emitters. Nanowire-based approaches provide a pathway to defect-free, highly efficient devices at nanoscale dimensions, while strain engineering, advanced templates, and buffer layer design enable improved indium incorporation and longer wavelength emission. Simultaneously, innovations in device architecture and fabrication processes are addressing challenges related to carrier dynamics, light extraction, and size scaling. Despite the remaining challenges, particularly in achieving high efficiency at deep-red wavelengths (>650 nm) and maintaining spectral stability under high injection conditions, the reported advances strongly support the feasibility of high-performance, full-color InGaN micro-LED displays in the next generation of red-light emission optoelectronic applications.

Table 2 summarizes the performance of representative green and red LEDs and LDs.

**Figure 14:**
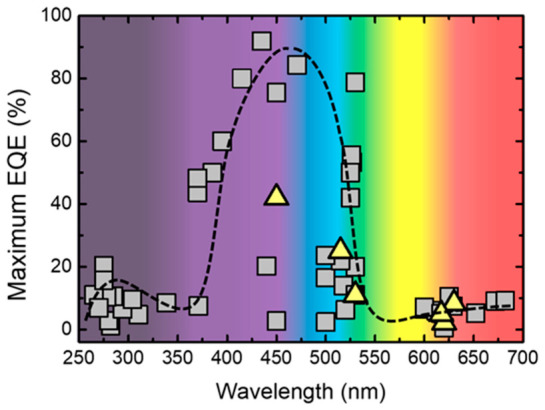
EQE as a function of emission wavelength for all the emitters discussed in the text. Squares refer to conventional emitters, triangle refers to micro-LEDs. The dashed line is a guide for the eye.

Figure 14 presents the EQE as a function of emission wavelength across the full optical spectrum for the emitters discussed. The highest efficiency is observed for blue emitters, with a decline toward the UV region (including a small peak reaching ~20% EQE in the UVB range) and a decrease at longer wavelengths, commonly known as the green gap and the “red edge.”

The EQE-wavelength behavior in the shorter wavelength range was discussed in detail in Section 3 and illustrated in Figure 9. The reduction in efficiency in the green–red region of nitride emitters is mainly attributed to difficulties in achieving higher In concentrations. Over time, various strategies aimed at overcoming these limitations have gradually contributed to “filling” the green gap and improving EQE values.

The challenges in the red region are similar but more pronounced. One promising approach is the development of red emitters based on micro- and nanostructures. Nevertheless, the EQE of red LEDs remains relatively low, and red laser diodes have not yet reached large-scale production.

## 6. Multicolor Emitters

### 6.1. White Emitters

White LEDs are widely used for LCD backlighting (monitors, TVs, and mobile devices) and in automotive applications such as headlights, daytime running lights, and interior illumination. They are typically based on blue InGaN emitters combined with phosphor emission. High-brightness white LEDs were first demonstrated in 1998 by Bando et al. [144], using a blue InGaN LED with a YAG:Ce phosphor.

**Figure 15:**
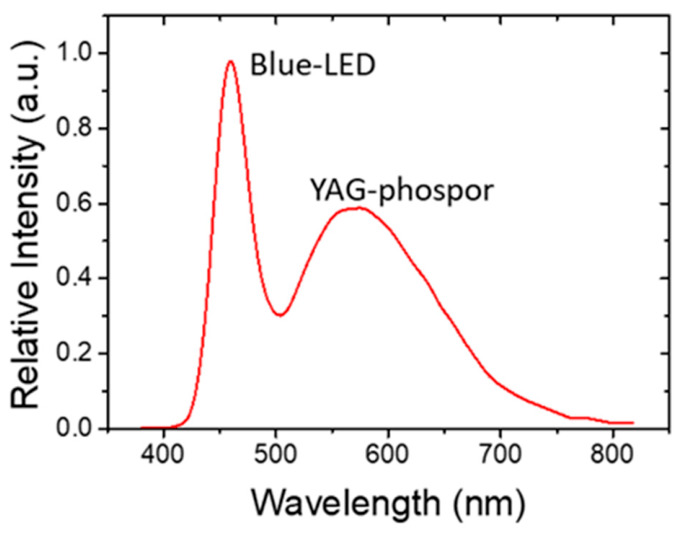
The exemplary spectrum of white LEDs.

An exemplary white LED spectrum (Figure 15) consists of a narrow blue emission band (~460 nm) and a broad green–yellow band (~555 nm), together covering the visible range from blue to red. The EQE of such devices produced at that time by the Nichia Corporation reached 7%.

Later developments significantly improved performance. In 2010, Narukawa et al. [45,46,47] demonstrated three types of white LEDs. The third was a high-power white LED with WPE = 58.5%, high luminous flux φv = 1913lm and luminous efficacy ηL = 135 lm/W, at a forward-bias current of 20mA. All devices were based on blue InGaN LEDs combined with different phosphor materials. The EQE of modern phosphor-converted white LEDs reaches ~40–60%, significantly higher than early devices, though still limited by conversion losses despite blue-emitter EQEs approaching ~90%.

### 6.2. RGB Emitters

To demonstrate the general concept of multicolor emitters, we consider the example of full-color monolithic InGaN micro-LEDs reported recently by Cheng et al. [145]. This approach enables the integration of red, green, and blue epitaxial layers within a single device, stacked using tunnel junctions (TJs) and grown entirely by MOVPE.

The red micro-LED exhibits an emission peak at approximately 650 nm at an injection current density of 1 A/cm^2^. As the injection current density increases, the emission peak undergoes a blue shift, moving from about 650 nm at 1 A/cm^2^ to 617 nm at 100 A/cm^2^. This corresponds to a relatively small wavelength shift of 33 nm. In contrast, the green and blue emitters show peak wavelengths of approximately 550 nm and 445 nm, respectively, which remain nearly independent of the driving current density.

**Figure 16:**
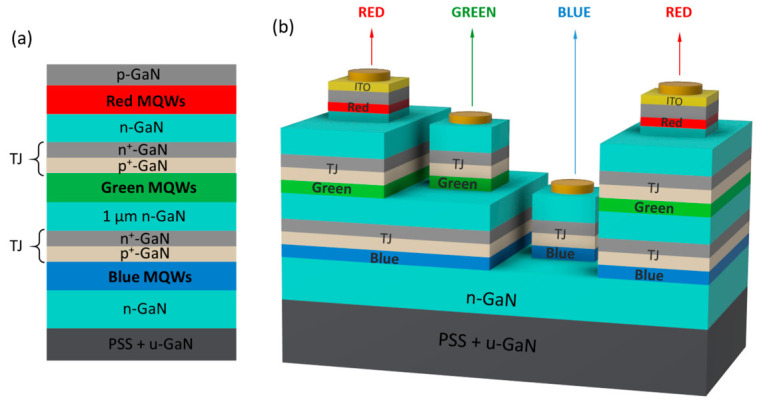
Schematic of (**a**) InGaN red, green, and blue MQW epitaxial structure, and (**b**) the three-dimensional structure of the monolithic InGaN micro-LED. TJ denotes the tunnel junction. Based on Figures 2a and 3c of Ref. [145].

The EQE of these devices reaches its peak at relatively low current densities. The peak EQE is about 0.3% for red emission, and approximately 17% for both green and blue emissions. The corresponding wall-plug efficiency (WPE) values are approximately 0.22% for red, 8% for green, and 10% for blue. Figure 16a illustrates a schematic of InGaN red, green, and blue epitaxial structures grown on a patterned sapphire substrate (PSS) using MOCVD. Figure 16b presents a schematic diagram of the three-dimensional structure of the monolithic InGaN micro-LED.

A self-aligned etching process is used, where both the top and bottom of the mesa reach the target layer at the same time. All mesa positions are defined during the first etching step. The subsequent second and third etching steps are used to define the green and blue LED mesas, as illustrated in Figure 16b. It is worth noting that the surface area assigned to each emission color varies depending on the required light intensity. Therefore, in the presented design, two separate red emitters are employed to compensate for their much lower emission efficiency. In contrast, the blue and green emitters exhibit higher and comparable intensities, allowing them to be realized as single emitters. The achievement of true red emission, along with wide color gamut coverage, underscores the significant potential of full-color monolithic InGaN micro-LEDs for use in display technologies.

## 7. Final Discussion

### 7.1. Performance Comparison of Nitride Emitters Across the Spectra Range

To facilitate direct comparison of device performance across different spectral regions, a more quantitative comparison is presented in Table 3 and Table 4, which summarize major milestones and representative performance metrics for nitride-based LEDs and laser diodes over the last three decades.

In LEDs, the first breakthrough came in 1994–1995 with the demonstration of high-brightness blue and green InGaN LEDs by Nakamura and co-workers, establishing III-nitrides as a practical optoelectronic material system. These achievements laid the foundation for the LED lighting revolution. A second major milestone was reached around 2010 through the introduction of transparent ITO contacts and patterned sapphire substrates (PSS), which significantly improved light extraction efficiency. As a result, blue LEDs achieved external quantum efficiencies approaching 80%, while white LEDs surpassed conventional fluorescent lamps in luminous efficacy, enabling widespread adoption of solid-state lighting.

Subsequent research expanded nitride technology into the ultraviolet region. Between 2014 and 2021, major advances in AlGaN materials and device design increased the efficiency of UV-C LEDs at 275–280 nm from about 7% to over 20%, opening new opportunities for sterilization and disinfection applications.

The most recent progress has focused on overcoming the long-standing issues of the green gap and “red gap.” Green LEDs and micro-LEDs have shown remarkable improvements, with EQE values exceeding 25% and, in some reports, approaching 80% at wavelengths around 530 nm. At the same time, the first highly efficient red InGaN LEDs have emerged, reaching EQEs above 10% at wavelengths of 625–680 nm. These developments are particularly important for full-color micro-displays and monolithic RGB light sources based entirely on the GaN material platform. Overall, the evolution of nitride LEDs illustrates a transition from improving blue-light emission and lighting efficiency toward extending operation across the entire visible and ultraviolet spectrum through advanced quantum-well engineering, strain management, and light-extraction technologies.

Table 4 highlights the rapid progress in nitride-based LDs over the past three decades, demonstrating significant improvements in performance, reliability, and wavelength coverage. The first violet nitride LDs, fabricated on sapphire substrates, operated only in pulsed mode and relied on gain-guided structures. Subsequent advances, including the introduction of ridge-waveguide and index-guided designs, enabled continuous-wave (CW) operation and substantially improved device stability. A major breakthrough was the extension of operational lifetimes from seconds to hundreds of hours and eventually to 10,000 h through the use of technologies such as epitaxial lateral overgrowth (ELOG) substrates and superlattice cladding layers.

The transition from sapphire to bulk GaN substrates further enhanced crystal quality and device reliability, leading to CW lifetimes exceeding 15,000 h. At the same time, nitride LD technology expanded beyond violet emission, enabling commercially important blue lasers around 450 nm and, later, green lasers near 515 nm. More recently, advances in Al-rich nitride materials have pushed laser operation into the deep-ultraviolet region, culminating in the demonstration of a 272 nm UVC laser on an AlN substrate.

Overall, these milestones illustrate the remarkable evolution of III-nitride laser technology, from proof-of-concept devices to highly reliable sources spanning the violet, blue, green, and ultraviolet spectral ranges.

### 7.2. Dominant Mechanisms Responsible for Efficiency Degradation

**Figure 17:**
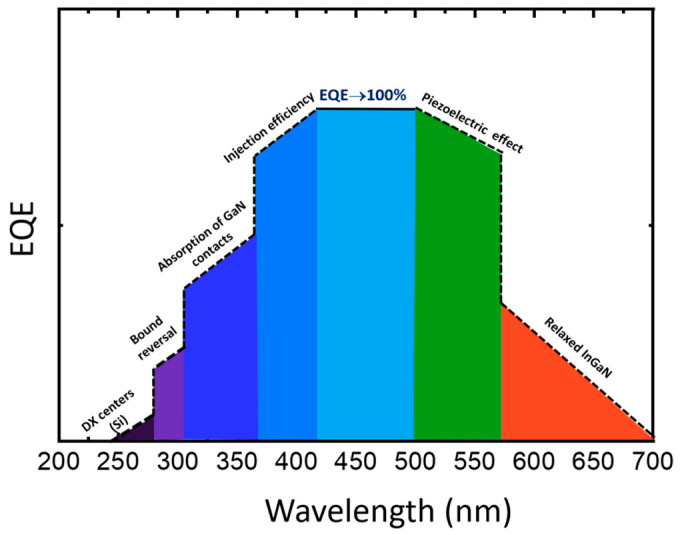
Schematic illustration of the relative importance of the dominant mechanisms responsible for efficiency degradation in III-nitride emitters, across different spectral regions.

The wavelength dependence of the EQE in III-nitride emitters arises from the interplay of several competing physical mechanisms. Figure 17 summarizes the dominant efficiency-limiting factors across the spectral range from the deep-UV to the red. The relative contribution of individual loss mechanisms varies with emission wavelength as a consequence of changes in alloy composition, carrier confinement, polarization effects, defect density, carrier transport, and light-extraction efficiency.

Toward the deep-UV region, efficiency is initially limited by poor carrier injection. At shorter wavelengths, additional losses arise from optical absorption in the GaN contact and cladding layers, which becomes increasingly significant for emission energies exceeding the GaN bandgap. Furthermore, band-order reversal in Al-rich AlGaN modifies the valence-band structure and the polarization characteristics of the emitted light, thereby reducing light-extraction efficiency. The formation of DX-like centers in highly Si-doped Al-rich AlGaN can further decrease the free-carrier concentration, increasing resistivity and degrading carrier injection.

For green and longer-wavelength emitters, the increasing levels of indium content required to reduce the bandgap result in a larger lattice mismatch between InGaN and GaN. This deteriorates crystal quality, increases non-radiative recombination, and promotes compositional inhomogeneity and indium segregation. In addition, strain relaxation alters the piezoelectric polarization fields and the QCSE, reducing the electron-hole wavefunction overlap. These effects collectively contribute to the reduction in radiative recombination efficiency observed in green emitters and are widely regarded as the primary origins of the so-called green gap. At even longer wavelengths, approaching the red spectral region, InGaN can no longer be grown pseudomorphically on GaN. The resulting strain relaxation and defect generation further enhance non-radiative recombination and lead to a pronounced reduction in device efficiency.

Overall, the evolution of EQE across the III-nitride spectral range cannot be attributed to a single mechanism. Rather, it reflects the combined influence of material quality, carrier transport, recombination dynamics, band-structure effects, and optical extraction processes, with the dominant limiting factors varying systematically from the deep-UV to the visible spectral regions.

### 7.3. Comparison of the Performance of InGaN- and AlGaN-Based Emitters

The performance of InGaN- and AlGaN-based emitters is governed not only by the emission wavelength, which is controlled by layer composition and thickness, but also by carrier injection, transport, and recombination processes within the active region. The internal quantum efficiency (IQE) is determined by the balance between radiative and non-radiative recombination rates. Photoluminescence (PL) measurements provide information on the intrinsic optical quality of the active region and the effectiveness of carrier confinement, whereas electroluminescence additionally reflects carrier injection efficiency, carrier transport across heterointerfaces, and current spreading within the device. Consequently, high-performance emitters require efficient carrier injection, strong carrier confinement, minimal leakage currents, and enhanced electron-hole wavefunction overlap, particularly in structures where polarization-induced electric fields influence carrier distribution and radiative recombination. The IQE of nitride quantum-well emitters is strongly affected by the structural quality, composition, and thermal stability of the active layers. Numerous studies employing X-ray diffraction (XRD), transmission electron microscopy (TEM), scanning electron microscopy (SEM), and PL spectroscopy have shown that InGaN quantum wells with indium contents of 18–25%, commonly used in blue and green emitters, are metastable and can undergo structural degradation during high-temperature processing.

**Figure 18:**
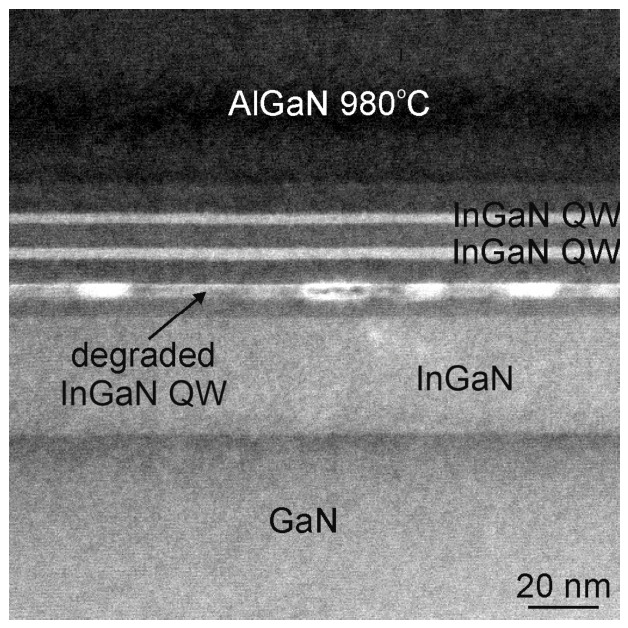
Scanning transmission electron microscopy (STEM) image showing the thermal degradation of a LD structure containing three In_0.15_Ga_0.85_N QWs grown on AlGaN and annealed at 980 °C. The first QW exhibits severe thermal degradation, with the formation of voids and indium-rich regions. Image provided by J. Smalc-Koziorowska; see Acknowledgments.

Smalc-Koziorowska et al. [153] demonstrated that thermal degradation of In-rich In_x_Ga_1−x_N quantum wells is driven by metal-vacancy diffusion and void formation, leading to strain relaxation, indium segregation, and alloy decomposition. These processes deteriorate the optical quality of the active region and contribute to efficiency losses in InGaN-based devices. Figure 18 illustrates these effects for a LD containing three In_0.155_Ga_0.85_N QWs. After annealing at 980 °C for 1 h in an ammonia atmosphere, pronounced interface roughening and the formation of void-like defects are observed within the first QW. These structural changes are accompanied by indium redistribution, phase separation, and increased compositional inhomogeneity, which promote carrier leakage and enhance non-radiative recombination, ultimately reducing the internal quantum efficiency (IQE). These findings highlight the importance of correlating optical performance with detailed structural characterization, particularly for high-indium-content InGaN layers required for efficient green-emitting devices.

Similarly, Lachowski et al. [154] showed that degradation of InGaN/GaN quantum wells above 900 °C is associated with the diffusion and clustering of gallium vacancies. By modifying vacancy concentrations through Si and Mg doping, they demonstrated that vacancies originating from the underlying GaN layer play a key role in the degradation process.

In contrast, AlGaN/GaN and AlGaN/AlGaN quantum wells generally exhibit significantly higher thermal and structural stability than InGaN/GaN quantum wells. Consequently, their thickness, composition, and interface quality can be controlled more reproducibly, resulting in a more direct relationship between structural quality and device performance [155]. An important exception occurs at very high Al contents, where increased strain, reduced p-type doping efficiency, alloy disorder, and defect formation become major factors limiting the efficiency of deep-ultraviolet emitters [156].

## 8. Future Challenges and Prospects

Looking ahead, several key challenges are expected to shape the development of nitride-based optoelectronics over the next decade. A major objective is to overcome the long-standing green gap and achieve highly efficient red-emitting InGaN devices, enabling a complete visible-spectrum technology platform. Significant improvements in UV-C LED performance are also required, with the goal of reaching efficiency levels comparable to those of visible LEDs. In the field of coherent light sources, the development of practical nitride-based VCSELs and novel laser architectures remains a priority. Another important direction is the realization of scalable quantum-photonic devices based on nitride quantum dots and nanostructures for emerging quantum technologies. Finally, the integration of nitride materials into monolithic full-color micro-LED displays and highly integrated photonic circuits is expected to play crucial roles in future display, communication, and sensing applications.

### 8.1. New Concepts

A key direction in the development of nitride emitters is improving emission efficiency, particularly at longer wavelengths. Achieving efficient true red emission remains challenging due to strain, defect formation, and phase separation at the higher indium contents. Ongoing efforts therefore focus on strain engineering (e.g., relaxed buffer layers) and alternative architectures, such as QDs and nanowires, which can better stabilize high-In compositions.

Mitigating the QCSE remains a key objective in nitride emitters. Traditionally, this has been addressed by reducing QW thickness; however, in a recent study, Muzioł [157] shows that the opposite approach, namely, using wider QWs, can also suppress QCSE. In such structures, ground-state transitions may be weakened, while excited-state transitions become dominant and highly efficient, leading to stronger overall emission than in conventional thin wells (<5 nm). This example illustrates how revisiting established design strategies can open new pathways for performance improvement.

Another key trend is improving efficiency at high current densities. Both LEDs and LDs suffer from efficiency droop, so work on future designs tends to focus on better carrier confinement, reduced Auger recombination, and improved electron–hole balance. This includes advanced QW designs, polarization engineering, and tunnel junctions to replace currently used resistive p-type layers.

Light extraction and optical design are also evolving. In the UV range, AlGaN-based emitters generally exhibit lower performance than their visible counterparts due to fundamental material and structural challenges, although steady progress continues. These limitations include high dislocation densities, poor p-type doping efficiency, and strong carrier localization effects, all of which reduce radiative recombination efficiency.

To address these challenges, advanced approaches such as polarization-induced doping are being actively developed, enabling efficient carrier injection without relying solely on conventional impurity doping. In III–nitride heterostructures (e.g., AlInN/GaN, AlGaN/GaN), polarization-induced charges at interfaces generate very high electron densities, leading to the formation of a two-dimensional electron gas (2DEG) even in the absence of intentional doping [158]. Additional strategies include improved heterostructure design, optimized electron-blocking layers, and the use of bulk AlN or low-defect templates to enhance crystal quality. Together, these efforts are expected to significantly improve the efficiency and reliability of AlGaN-based UV emitters.

On the fabrication side, significant advances are being made to further enhance device performance. For LEDs, photonic crystals, surface texturing, and micro-/nano-patterned substrates are being refined to increase light extraction and push EQE to higher levels. Monolithic integration is also a major focus, particularly for micro-LED displays, where RGB emitters are integrated on a single chip, as well as for systems combining light emitters with driving electronics.

For LDs, new device concepts are emerging within photonic integrated circuits (PICs), enabling the integration of multiple optical and electronic functions on a single platform. In particular, vertical-cavity surface-emitting lasers (VCSELs) and photonic crystal surface-emitting lasers (PCSELs) are attracting increasing attention. VCSELs are already well developed, a category representing technologically advanced and highly sophisticated devices, with commercial examples such as lasers developed by Nichia and Sony.

Conventional edge-emitting lasers do not meet the requirements of all applications. In this context, PCSELs are considered promising candidates for future high-power light sources and may eventually complement or replace edge-emitting LDs in selected applications.

In the broader context of integration, an open question is whether it is advantageous to combine not only optical but also electronic functionalities within a single structure. One proposed approach is the fabrication of light emitters on one side of a wafer and electronic components, such as transistors, on the opposite side. This concept, referred to as “dualtronics” [159], demonstrates the feasibility of integrating photonic devices on the cation face and electronic devices on the anion face of the same polar semiconductor wafer. Such an approach opens new possibilities for utilizing both faces of a single structure, enabling the simultaneous implementation of electronic, photonic, and even acoustic functionalities, and thereby significantly enhancing the capabilities of nitride-based semiconductor technologies.

In summary, the field is advancing toward improved material control, innovative device architectures, and increasingly integrated optoelectronic systems.

### 8.2. Micro-LED Technology

Micro-LEDs are among the most promising technologies for next-generation displays, particularly AR/VR systems. Compared with conventional broad-area MQW LEDs, μLEDs promise greater brightness, improved current-density operation, lower power consumption, faster modulation speeds, and excellent scalability for ultra-high-resolution displays and CMOS-integrated optoelectronic systems [125,160,161]. Recent studies have shown that improvements in light extraction, emission directionality, sidewall passivation, and optimized InGaN QW designs can, in some cases, enable highly efficient red InGaN μLEDs [125,162].

Thermal management also becomes increasingly important at the high current densities typical of μLED operation. Substrate engineering, advanced interconnects, and CMOS-compatible integration strategies have been shown to significantly improve thermal performance, optical stability, and device reliability [163,164].

From a device-performance perspective, however, miniaturization introduces challenges that are absent or less pronounced in conventional MQW structures. As device dimensions decrease, carrier diffusion, sidewall defects, plasma-induced damage, and surface recombination increasingly influence electrical and optical performance. These effects can increase leakage currents, enhance Shockley–Read–Hall (SRH) recombination, and reduce EQE [161,165,166].

Overall, μLEDs provide clear advantages over conventional MQW LEDs in terms of modulation speed, integration capability, and display resolution. However, their performance remains strongly dependent on carrier transport, sidewall quality, thermal management, and fabrication technology, making continued optimization of materials, device design, and integration processes essential for realizing their full potential.

### 8.3. Semipolar Orientations

Semipolar InGaN-based light emitters have been widely investigated because their reduced polarization fields lead to improved electron-hole wavefunction overlap, higher radiative recombination rates, reduced efficiency droop, easier realization of long-wavelength (green/yellow) emitters, and lower threshold currents in laser diodes.

However, their practical implementation remains limited by high oxygen incorporation during growth [18,19], strain relaxation through dislocation formation [19], growth complexity, and the limited availability of large-area, high-quality substrates. Consequently, despite their clear physical advantages, the performance and efficiency of nonpolar and semipolar LEDs and LDs have generally remained below initial expectations because of material and structural limitations [15].

Currently, the prospects of semipolar nitride emitters are primarily associated with polarization engineering and the enhanced optical performance offered by columnar and nanostructured geometries. These structures facilitate the growth of high-quality semipolar facets while providing additional benefits related to strain relaxation and improved light extraction. Among the various approaches, two-dimensional (2D) columnar and nanocolumn structures have emerged as promising platforms for the successful growth and realization of semipolar emitters [167].

Recently published studies on semipolar emitters have increasingly focused on micro-LED architectures. For example, Inaba et al. [168] investigated emission-plane control in GaInN/GaN multiple-quantum-well (MQW) shell nanowire LEDs. They systematically examined the emission characteristics of nanowire LEDs with different p-GaN shell growth times and emission areas. The results demonstrated that suppression of emission from the c-plane (0001) apex region is essential for achieving high device efficiency. Minimizing the area of the grown (0001) facet and promoting carrier injection into the semipolar sidewall facets were identified as effective approaches for enhancing light output. These findings indicate that controlling the emission planes of nanowires through structural design and growth optimization is highly relevant for the development of efficient nanowire-based micro-LEDs. Overall, engineering the nanowire morphology through the incorporation of superlattice structures, together with tuning the current-injection pathways by adjusting the p-GaN shell thickness and emission area, represents an important strategy for next-generation micro-LED technologies.

Other recent examples include the work of Li et al. [169], who investigated semipolar (11̅22) InGaN/GaN SL templates grown on m-plane sapphire by MOCVD. They showed that strain relaxation through one-dimensional misfit dislocation arrays improved surface morphology and enhanced indium incorporation, although excessive relaxation degraded template quality.

Hu et al. [170] (2024) demonstrated semipolar (10̅13) InGaN/GaN LEDs with an IQE approaching 86% at 460 nm. The reduced polarization fields enabled improved electron-hole overlap and high efficiency at elevated current densities, highlighting the potential of semipolar structures for micro-LED applications.

Xing et al. [171] (2025) reported 633 nm InGaN-based red LEDs on semipolar (11̅22) GaN templates. The devices exhibited reduced QCSE, lower efficiency droop, and smaller wavelength shifts compared with conventional polar LEDs, supporting semipolar orientations as a promising route toward monolithic phosphor-free RGB emitters.

Gandrothula et al. [172] demonstrated pulsed laser operation in semipolar GaN laser diodes at 428 nm with a threshold current density of approximately 20 kA cm^−2^, confirming the feasibility of semipolar orientations for laser emitters.

Furthermore, Ou et al. [173] reported semipolar InGaN microcavity LEDs exhibiting polarized emission, demonstrating additional functionalities of semipolar emitters for advanced display and photonic applications.

### 8.4. Hexagonal Boron Nitride

In recent years, a new class of layered materials has emerged that can be exfoliated into atomically thin layers, stacked into heterostructures, and grown on in wafer-scale applications. Among them, hexagonal boron nitride (hBN), a van der Waals material with a wide bandgap (~6 eV), offers several advantages over conventional bulk semiconductors. Its atomically flat surface and low defect density make it an excellent substrate and dielectric layer for 2D heterostructures. hBN can also function as an insulating or active layer in devices such as LEDs, photodetectors, and HEMTs. Recent advances in III-nitride epitaxy have enabled wafer-scale growth on sapphire, AlN, and SiC substrates, while its strong optical properties, large exciton binding energy (~0.7 eV), and unique thermal characteristics make it attractive for optoelectronic applications. Furthermore, Mg doping has enabled p-type conductivity, supporting the development of hBN/AlGaN p-n junctions.

Zaiter et al. [96] (2023) demonstrated deep-UV emission (275–280 nm) from MBE-grown AlGaN quantum dots on h-BN, highlighting the potential of hybrid h-BN/nitride structures.

Beyond conventional optoelectronic applications, hBN has attracted considerable interest as a platform for quantum photonics due to its ability to host single-photon emitters operating in the UV, blue, and visible spectral ranges. This makes hBN relevant for applications beyond conventional optoelectronics, including quantum communication and cryptography, quantum computing, quantum sensing and metrology, and integrated quantum photonic circuits.

Recent reviews [174,175] summarize advances in h-BN for quantum photonics, covering material growth, defect engineering, and device integration. They highlight the potential of h-BN for light-emitting devices and as a platform for single-photon sources. Attention has been given to spin-active defects in h-BN, which can act as bright single-photon emitters (SPEs) and have attracted significant interest for quantum technologies.

Considerable progress has been made in the controlled creation, stabilization, and integration of these emitters with scalable photonic resonators, although the field remains at an early stage. In contrast, ultraviolet emitters (~4.1 eV) have been studied mainly as ensembles, with only a single report of single-photon emission to date [176].

In addition, blue-emitting defects can be created on demand in large-area, high-quality hBN flakes. In 2021, Fournier et al. [177] reported single-photon emitters in high-purity synthetic hBN with an emission wavelength of 435 nm that can be activated at selected locations using an electron beam. The emitters can be positioned with subwavelength spatial accuracy, making them suitable for integration into optical microstructures. While antibunching is preserved up to room temperature, the emission loses coherence at elevated temperatures. Nevertheless, these results highlight the potential of engineered quantum emitters in hBN for future quantum photonic applications based on 2D materials.

Most research has focused on visible defects in hBN, because of their high brightness and abundance. These defects exhibit stable single-photon emission up to room temperature and show highly reproducible emission wavelengths, with much smaller spectral variation than in epitaxial hBN. Such properties are important for the development of integrated quantum photonic devices based on identical emitters in 2D materials.

hBN has emerged as a promising platform for quantum photonics due to its room-temperature single-photon emitters and successful integration with photonic waveguides and circuits, achieving coupling efficiencies of up to ~40%. However, several challenges remain for scalable technologies, including precise control of emitter position, orientation, and emission wavelength, identification of their atomic structure, realization of stable emitters in monolayer hBN, and reproducible, wafer-scale fabrication. Further advances in defect engineering, cavity coupling to enhance brightness, and electrically driven single-photon emission will be crucial for the development of scalable hBN-based quantum photonic devices.

Improving growth techniques and defect control is essential for optical, electronic, neutron-detection-based, and quantum photonic applications. Recent advances in high-pressure growth have produced highly uniform h-BN crystals with excellent structural quality and thicknesses up to ~30 μm [178]. In addition, exfoliated h-BN flakes used in graphene heterostructures enable carrier mobilities exceeding 21.2 m^2^·V^−1^·s^−1^ at 230 K, highlighting the material’s potential for advanced electronic and optoelectronic devices. Continued progress in crystal growth and defect engineering will be crucial for scalable hBN-based quantum technologies.

### 8.5. Nitride/Oxide Heterostructures

Nitride/oxide heterostructures, in both WZ and RS configurations, represent a promising platform for high-efficiency UV emitters, photodetectors, HEMTs, and multifunctional devices. By combining III-nitrides (GaN, AlGaN, and InGaN) with oxides such as MgO, BaTiO_3_, or ZnO-based alloys, they integrate the advantages of both material systems, combining optoelectronic functionality with dielectric, ferroelectric, and magnetic properties, extending device capabilities beyond those of conventional nitride technologies. Although interface engineering remains a significant challenge, numerous experimental studies have already demonstrated the feasibility and considerable potential of these hybrid systems.

Nitride/oxide hybrid systems are often more attractive for photodetectors than for emitters because their main advantage arises from the heterointerface. Combining nitrides (GaN, AlGaN, and InN) with oxides (ZnO, TiO_2_, β-Ga_2_O_3_, and SnO_2_) can create built-in electric fields that efficiently separate photogenerated electrons and holes, reduce carrier recombination losses, and enhance charge collection. These properties are highly desirable for UV photodetectors.

In contrast, efficient light emitters require electrons and holes to be confined in the same region and recombine radiatively with high probability. Nitride/oxide interfaces often introduce lattice mismatch, interface defects, and trap states that act as non-radiative recombination centers, reducing emission efficiency. Therefore, unlike pure nitride semiconductors, which are widely used in LEDs and laser diodes, nitride/oxide hybrid structures generally offer greater advantages for photodetection than for light emission. A notable exception is the ZnO/GaN system.

#### 8.5.1. ZnO/GaN

ZnO and GaN have similar crystal structures, relatively small lattice mismatch, and comparable band gaps. Because of this, they have been investigated for both LEDs and photodetectors. However, despite decades of research, ZnO/GaN structures have generally achieved greater success and broader interest as photodetectors and sensors than as commercially competitive LEDs.

ZnO/GaN is the most mature nitride/oxide heterojunction, with a coherent wz interface supporting strong excitonic recombination. The ZnO/GaN heterostructures have evolved from early blue and white LED demonstrations to engineered nanoscale emitters and ultrafast UV detectors. The coherent hexagonal interface, strong excitonic effects, and polarization-induced 2DEG formation make this system a benchmark for oxide/nitride integration and a reference point for designing next-generation optoelectronic and photonic devices.

Between 2005 and 2025, this approach has enabled LEDs and self-powered photodetectors. Early work by Hwang et al. [179] (2005) demonstrated a p-ZnO/n-GaN LED (409 nm, 5.4 V), followed by UV emitters reported by Chuang et al. [180] (2007), with emission around 385 nm, and by Chen et al. [181] (2009), with emission near 415 nm. Broadband emission, including white light (450/560 nm), was achieved by Sadaf et al. [182] (2010).

In 2013, Wei et al. [183] demonstrated that introducing a GaN buffer layer significantly improves the structural and optical quality of ZnO thin films grown on Si substrates. The GaN interlayer promotes epitaxial growth, changes the ZnO growth mode from one-dimensional to two-dimensional, reduces defect-related emissions, and enhances UV luminescence. These findings highlight the importance of interface engineering in ZnO/GaN heterostructures and support their potential for high-efficiency UV optoelectronic devices and silicon-compatible integration. More recent studies include the optimized LEDs (~395 nm, ~3 V) reported by Macaluso et al. [184] (2020).

**Figure 19:**
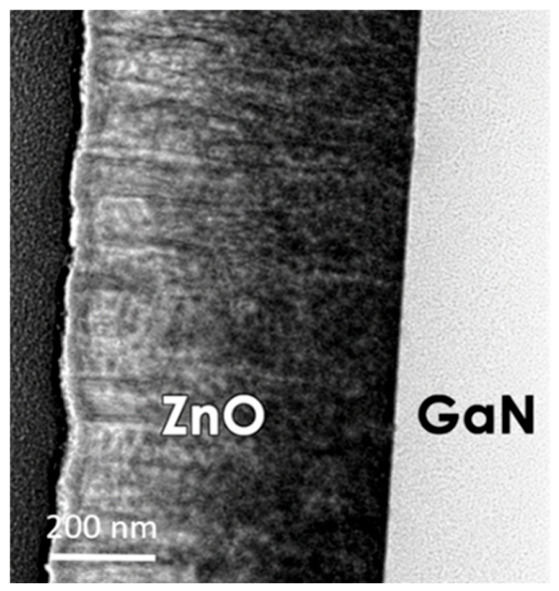
TEM image of the ZnO/GaN interface. Reproduced from Ref. [185] with permission from the American Chemical Society. Copyright © 2024, American Chemical Society.

High-speed self-powered UV photodetectors were reported by Kaur et al. [185] in 2024. The interface quality is evidenced in Figure 19, which shows a cross-sectional transmission electron microscopy (TEM) image of the sharp contrast at the junction between the two materials, which suggests an abrupt interface. A smooth transition of the atomic arrangements at the interface is very clear, which is consistent with the presence of very little lattice mismatch between the two materials.

#### 8.5.2. ZnGeN_2_/GaN

Another example of a hybrid oxide-like/nitride-derived structure was reported in 2024 by Miller et al. [186], who demonstrated the MBE growth of ZnGeN_2_/GaN SLs for color-mixed LEDs and mitigation of the green gap. ZnGeN_2_, which is nearly lattice-matched to GaN, can be integrated into existing III-N device architectures and is predicted to increase spontaneous emission rates by nearly a factor of five compared with conventional InGaN emitters. The authors identified Ga–Ge interdiffusion as a key issue limiting luminescence efficiency but showed that optimized growth conditions significantly improved optical quality. The resulting SLs exhibited enhanced emission characteristics, marking an important step toward efficient hybrid III-N/II-IV-N_2_ optoelectronic devices and high-performance color-mixed LEDs.

#### 8.5.3. GaN/MgO

GaN/MgO is promising system for dielectric integration and phase engineering, offering low interface state density and access to cubic nitrides with reduced polarization fields. It supports improved device performance, although challenges such as thermal instability and interdiffusion remain. Recently, Luna et al. [187] demonstrated that MgO(001) substrates enable the epitaxial growth of high-quality cubic III-nitride films, with buffer layers playing a crucial role in suppressing defects and stabilizing the cubic phase. Their work highlights the importance of interface engineering and precise growth control for overcoming fabrication challenges in GaN/MgO heterostructures. The compatibility of MgO with cubic nitrides and the resulting tunable optoelectronic properties support the scalability and future integration of these heterojunctions in advanced optoelectronic and photovoltaic devices.

#### 8.5.4. Oxide/GaN Photodetectors

In 2025, Han et al. [188] demonstrated a high-performance UV photodetector based on a β-Ga_2_O_3_/GaN heterojunction formed by thermally oxidizing GaN. Higher-quality GaN templates produced smoother β-Ga_2_O_3_ layers with fewer oxygen vacancies, leading to excellent device performance, including responsivity up to 2493.5 A/W, detectivity above 10^16^ Jones, and a rise time of ~0.27 ms. Self-powered solar-blind operation with response times as short as 5 µs was also achieved. These results highlight the importance of GaN template quality and demonstrate controlled oxidation as a practical route for integrating high-quality Ga_2_O_3_ with GaN in next-generation deep-UV photodetectors.

**Figure 20:**
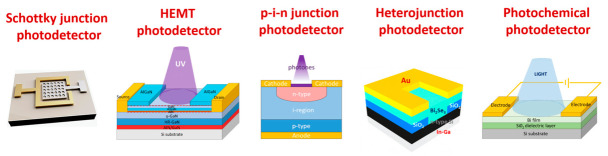
Schematic illustration of selected types of nitride/oxide-based photodetectors. Based on Figure 1, Ref. [189].

In a 2025 review, Zhang et al. [189] highlighted that the performance of Oxide/GaN photodetectors depends strongly on interface engineering, defect passivation, and scalable fabrication. Integrating oxide layers with GaN improves charge separation, spectral response, and detectivity, demonstrating the promise of oxide/nitride heterojunctions for advanced optoelectronic devices. GaN-based photodetectors outperform conventional Si devices, particularly in the UV range, and can be realized as Schottky, heterojunction, p-i-n, HEMT-based, or photoelectrochemical structures. Figure 20 presents schematic examples of these device architectures.

#### 8.5.5. Ferroelectric/Oxide/Nitride Heterostructures

One representative ferroelectric/oxide/nitride heterostructure is BaTiO_3_/MgO/AlGaN/GaN/Si. Li et al. [190] showed that the switchable polarization of BaTiO_3_ modulates the 2DEG at the AlGaN/GaN interface, enabling threshold-voltage control and non-volatile memory operation. The MgO interlayer acts as a lattice-matching and diffusion-barrier layer, demonstrating the potential of such heterostructures for multifunctional GaN-based electronics.

#### 8.5.6. Challenges and Prospects in Nitride/Oxide Systems

The development of nitride/oxide Systems is constrained by challenges associated with lattice mismatch, crystal-symmetry differences, thermal-expansion mismatch, and chemical intermixing, all of which complicate the formation of high-quality interfaces.

In successful fabrication of nitride/oxide emitters, achieving defect-free nitride/oxide interfaces is particularly challenging because nitrides and oxides often require different growth conditions. Even relatively compatible systems such as ZnO/GaN can develop strain-induced defects, while more dissimilar systems, such as MgO/GaN, face greater interface-quality issues. Growth-temperature compatibility is also critical, as thermal stress, interdiffusion, and compositional changes can degrade structural and electrical properties. Precise stoichiometry control is especially important in complex materials such as ZnGeN_2_. As a result, advanced deposition techniques, including MBE, MOCVD, PLD, and ALD, are frequently required. Fischer et al. [191] (2024) further demonstrated that the properties of MgO strongly depend on deposition temperature: low-temperature growth produced oxygen-rich semiconducting and ferromagnetic films, whereas RT growth yielded stoichiometric MgO with excellent dielectric properties. This highlights the importance of growth conditions in tailoring oxide/nitride heterostructures.

Interface quality plays a central role in carrier transport and recombination. Defects such as dangling bonds and vacancies can act as non-radiative recombination centers and increase leakage currents. Efficient device operation therefore requires careful band-alignment engineering, interface passivation, and control of spontaneous and piezoelectric polarization fields. While wide-bandgap oxides such as MgO provide excellent carrier confinement, excessively large band offsets may impede carrier transport.

Although many nitride/oxide devices demonstrate excellent laboratory performance, large-scale manufacturing remains challenging. The industrial maturity of GaN technology provides a strong platform for oxide integration. ZnO/GaN systems are particularly attractive, because ZnO can be deposited over large areas using cost-effective methods such as sputtering, solution processing, and ALD. However, challenges related to wafer-scale reproducibility, defect control, interface uniformity, and long-term reliability still need to be addressed. More complex materials such as ZnGeN_2_ also present synthesis and cost challenges that may limit commercialization.

Overall, nitride/oxide heterostructures remain a promising platform for high-efficiency UV emitters, photodetectors, and multifunctional devices combining optoelectronic, dielectric, ferroelectric, and magnetic functionalities. By integrating III-nitrides with functional oxides, they offer capabilities beyond those of conventional nitride technologies and hold significant potential for applications in UV photonics, power electronics, spintronics, and advanced sensing. However, their development is still constrained by lattice mismatch, crystal-symmetry differences, thermal-expansion mismatch, chemical intermixing, and the difficulty of achieving high-quality interfaces. Continued advances in epitaxial growth, interface engineering, and defect control are expected to accelerate their practical implementation.

## 9. Summary

This review summarizes recent progress in light emitters based on nitride quantum structures, with particular emphasis on the evolution of EQE. These devices are primarily based on InGaN QWs, with AlGaN used for UV light emission. Their performance is governed by intrinsic material properties, leading to strong wavelength dependence. Blue InGaN LEDs (~450 nm) achieve the highest efficiencies (~70–80% EQE). Toward shorter wavelengths, UV AlGaN emitters remain less efficient due to material limitations, although steady progress is being made. Toward longer wavelengths, efficiency decreases due to difficulties in growing high-quality, high-indium InGaN QWs. In the green spectral region (“green gap”), EQE is typically reduced to ~20–35%, dropping further to ~5–10% in the amber–red range. Notably, efficient red nitride laser diodes have not yet been realized.

Key mechanisms limiting EQE and strategies for improvement are discussed. Emerging technologies, such as micro-LEDs and alternative emitter concepts, offer potential for further performance improvements, although large-scale deployment is still limited. Future advances are expected to arise from improved control of carrier transport and recombination, continued progress in active-region and heterostructure engineering, and the integration of emerging material platforms for optoelectronic and quantum-photonic applications.

## Figures and Tables

**Table 1:** Comparison of different nitride blue, violet and UV light emitters.

Characteristic	Wavelength (nm)	EQE (%)	Year/Reference
**Violet/Blue**			
Zn-doped InGaN/GaN LEDs	450	2.7	1994 Nakamura [43]
2 nm thick InGaN/GaN SQW LEDs	500	2.4	1994 Nakamura [44]
LEDs with optimized IQE and light extraction (ITO p-electrode and PSS)	450 471	75.5 84.3	2006–2010 Narukawa [45,46,47]
LEDs with flip-chip architecture	415	80	2015 Hurni [48]
Cascaded micro-LEDs	450	42	2021 Li [49]
Nano-LEDs: sol–gel SiO_2_ surface passivation	440	20.2	2022 Sheen [50]
In_0.15_Ga_0.85_N/In_0.02_Ga_0.98_N LEDs	435	91.9	2024 Choi [51]
In_0.15_Ga_0.85_N/In_0.02_Ga_0.98_N LDs *	440	44.7	2024 Choi [51]
**UVA**			
InGaN/AlGaN (In ~ 0) LEDs	371	7.5	1998 Mukai [55]
LEDs with improved growth & technology	385	49.8	2014 Muramoto [56]
Al_0.06_Ga_0.94_N/Al_0.16_Ga_0.84_N MQW LDs *	338.6	8.5	2016 Taketomi [65]
Vertical LEDs with in situ AlN and ex situ AlGaN nucleation layer	370	43.7 48.2	2018 Oh [57]
InGaN/GaN/AlGaN/GaN optimized LEDs	395	60	2020 Li [60]
**UVB**			
InAlGaN-based LEDs with high Al content	282	1.2	2009 Hirayama [72]
LEDs with high-crystal-quality AlN templates	280	2.78	2010 Fujioka [74]
LEDs with optimized carrier transport and AlGaN MQW design	310	4.7	2020 Khan [75]
LEDs with increased Al content in AlGaN MQW	294	6.5	2020 Khan [75]
Germicidal UV LED with heavily Si-doped n-AlGaN MQWs	285	10.6	2023 Wang [77]
Ring-shaped micro-LEDs	280	6.17	2024 Zhao [79]
LEDs with photonic crystal and nano-patterned substrates	304	9.6	2025 Khan [76]
**UVC**			
Optimized chip encapsulation	278	10.4	2012 Shatalov [84]
Improved growth, enhanced light extraction	279	7.0	2014 Hirayama [85]
AlN template on PSS	275	20.3	2017 Takano [87]
Optimized reflective p-electrodes	279	9.0	2018 Maeda [88]
AlGaN/GaN/AlGaN tunnel junction	265	11	2020 Pandey [89]
Nano-patterned light extraction	273	5.19	2021 Zheng [90]
p-layer optical optimization	275	15.7	2021 Matsukura [91]
Optimized MQWs and tunneling junction	270	6.9	2024 Liu [92]

* For LDs, differential EQE is usually used.

**Table 2:** Comparison of different green–red nitride emitters.

Characteristic	Wavelength (nm)	EQE (%)	Year/Reference
**Green**			
p-AlGaN/InGaN/n-GaN MQWs	520	6.3	1995 Nakamura [113]
InGaN/GaN MQWs on c-plane patterned sapphire	527	53.3	2018 Li [114]
Optimized V-pits in InGaN/GaN MQWs	525	42	2018 Zhou [115]
Optimized InGaN/GaN MQWs on PSS	526	55.6	2019 Lv [116]
AlGaN interlayers, Si substrate.	525	50	2020 Guo [117]
In_0.25_Ga_0.75_N/In_0.02_Ga_0.98_N LEDs	530	78.8	2024 Choi [51]
In_0.25_Ga_0.75_N/In_0.02_Ga_0.98_N LDs	500	23.6	2024 Choi [51]
Cascaded μLEDs	518	14	2021 Li [49]
Nanowire LEDs grown by PAMBE	530	11.0	2022 Liu [123]
Submicron-scale μLEDs	515	25.2	2023 Pandey [124]
50 μm μLEDs	500	16.5	2024 Smith [125]
Commercial LEDs	520–540	15–35	
Commercial LDs	510–530	20–25	
**Yellow–Amber**			
Hybrid MQW structures	620	0.6	2016 Iida [131]
μLEDs based on QDs	617	4.9	2022 Yu [132]
Stress relaxation + high carrier injection	617	5.11	2022 Horng [133]
V-defect-engineered LEDs	600	6.5	Ewing [134]
QW engineering	612	6.0	2023 Li [135]
1 μ μLEDs	600	7.1	2024 Smith [125]
**Red**			
Device-level engineering	652	5.2	2022 Huang [137]
N-polar InGaN nanowires	620	2.2	2022 Pandey [138]
Nanowire LEDs with optimized Mg doping	630	8.3	2023 Pandey [139]
Grain coalescence in the composite buffer	629	7.4	2023 Chen [140]
Planar InGaN LED-band engineering	625	10.5	2023 Lee [141]
GaN on columnar structures on porous SiN	672	9.1	2024 Xing [142]
GaN on columnar structures on porous SiN improved	682	9.2	2025 Xing [143]

**Table 3:** Performance milestones of nitride LEDs.

Spectral Range	Characteristic	Wavelength nm	Key Performance	Year, Reference
Blue	Zn-doped InGaN/AlGaN double heterostructures	450	Luminous intensity ~ 1 cd EQE = 2.7%	1994 [43]
Green	p-AlGaN/InGaN/n-GaN MQWs	520	EQE = 6.3%	1995 [113]
Blue	ITO p-electrode, PSS	471	P_out_ = 47 1mW EQE ~80%	2010 [47]
White	Superior to 20 W class fluorescent lamp	-	WPE *=* 58.5% φ_v_ = 1913 lm $\eta_{L}$ = 135 lm W^−1^ at 20mA	2010 [47]
UV	InAlGaN/InAlGaN three-layer MQWs	220–350	EQE = 7% for 279 nm	2002 [146] 2014 [85]
UVC	Al_0.65_Ga_0.35_N:Mg contact layer	275	EQE = 20.3% at 20-mA P_out_ = 18.3	2017 [87]
UVC	p-layer optical optimization	275	P_out_ = 385mV (15,000 mA) WPE = 15.3 (10.5 mA), EQE = 15.7%	2021 [91]
Green	InGaN/GaN MQWs on c-plane patterned sapphire	527	EQE = 53.3%, WPE = 54.1%, ηv = 329 lm/W	2018 [114]
Green	Nanowire LEDs grown by PAMBE	530	EQE = 11.0%	2022 [123]
Green	Submicron-scale μLEDs	515	EQE = 25.2%, WPE = 20.7%	2023 [124]
Red	InGaN QWs with GaN/AlGaN barriers	625	EQE = 10.5% (at 10 A/cm^2^)	2023 [141]
Green	50 μm μLEDs	500	EQE = 16.5%	2024 [125]
Green	In_0.25_Ga_0.75_N/In_0.02_Ga_0.98_N MQWs	530	EQE = 78.8%	2024 [51]
Red	GaN on columnar structures on porous SiN	682	EQE = 9.2%	2025 [143]

**Table 4:** Performance milestones of nitride LDs.

Milestone	Substrate	Wavelength (nm)	Operation Mode/Lifetime	Features	Ref.
First nitride-based LD-violet	Sapphire	417	Pulse	Gain-guided	[52]
Ridge waveguide	Sapphire	411	Pulse	Index-guided	[147]
CW operation	Sapphire	405	CW (1 s)	Index-guided	[53]
CW-longer lifetime	Sapphire	416	CW (300 h)	Index-guided, p-down mounted	[148]
10,000 h lifetime	Sapphire		CW (10,000h)	Gain guided, ElOG substrate, SL cladding	[149]
Bulk GaN substrate	GaN	405	CW (15,000 h)	ELOG on GaN	[150]
Blue laser diode	GaN	450	CW	ELOG on GaN	[151]
Green laser	GaN	515	CW	GaN	[152]
UVC laser	AlN	272	Pulse	Polarization doped p-type	[80]

## Data Availability

No new data were created or analyzed in this study. Data sharing is not applicable to this article.
